# Energy-Efficient Clustering Mechanism of Routing Protocol for Heterogeneous Wireless Sensor Network Based on Bamboo Forest Growth Optimizer

**DOI:** 10.3390/e24070980

**Published:** 2022-07-15

**Authors:** Qing Feng, Shu-Chuan Chu, Jeng-Shyang Pan, Jie Wu, Tien-Szu Pan

**Affiliations:** 1 College of Computer Science and Engineering, Shandong University of Science and Technology, Qingdao 266590, China; 202082060009@sdust.edu.cn (Q.F.); jspan@cc.kuas.edu.tw (J.-S.P.); 2 Department of Information Management, Chaoyang University of Technology, Taichung 41349, Taiwan; 3 School of Electrical and Information Engineering, Zhengzhou University of Light Industry, Zhengzhou 450002, China; wujie@zzuli.edu.cn; 4 Department of Electronic Engineering, National Kaohsiung University of Science and Technology, Kaohsiung 82445, Taiwan; tpan@nkust.edu.tw

**Keywords:** wireless sensor networks, energy-efficient clustering mechanism, bamboo forest growth optimizer

## Abstract

In wireless sensor networks (WSN), most sensor nodes are powered by batteries with limited power, meaning the quality of the network may deteriorate at any time. Therefore, to reduce the energy consumption of sensor nodes and extend the lifetime of the network, this study proposes a novel energy-efficient clustering mechanism of a routing protocol. First, a novel metaheuristic algorithm is proposed, based on differential equations of bamboo growth and the Gaussian mixture model, called the bamboo growth optimizer (BFGO). Second, based on the BFGO algorithm, a clustering mechanism of a routing protocol (BFGO-C) is proposed, in which the encoding method and fitness function are redesigned. It can maximize the energy efficiency and minimize the transmission distance. In addition, heterogeneous nodes are added to the WSN to distinguish tasks among nodes and extend the lifetime of the network. Finally, this paper compares the proposed BFGO-C with three classic clustering protocols. The results show that the protocol based on the BFGO-C can be successfully applied to the clustering routing protocol and can effectively reduce energy consumption and enhance network performance.

## 1. Introduction

Guided by the development trend of the “sensor city”, wireless sensor networks (WSN) have been widely used in the field of information, from the field of military and national defense to the fields of medical care, industry and agriculture, urban management, environmental monitoring [1], and smart homes [2] that are closely related to people. The WSN is an ad hoc network composed of randomly distributed sensor nodes [3]. The nodes perceive the environment to enable data collection, processing and transmission. However, the sensor nodes in WSN use limited energy (such as batteries), and in some complex working environments, it is difficult to supply power in time, which will lead to the unreliable lifetime and quality of the network. In addition, for scenarios with high real-time requirements, such as disaster monitoring, military supervision, medical inspection, etc., it is even more necessary to consider how to balance the energy consumption of nodes [4]. Therefore, how to balance the energy consumption and extend the lifetime of network is the research focus in WSN.

**Figure 1:**
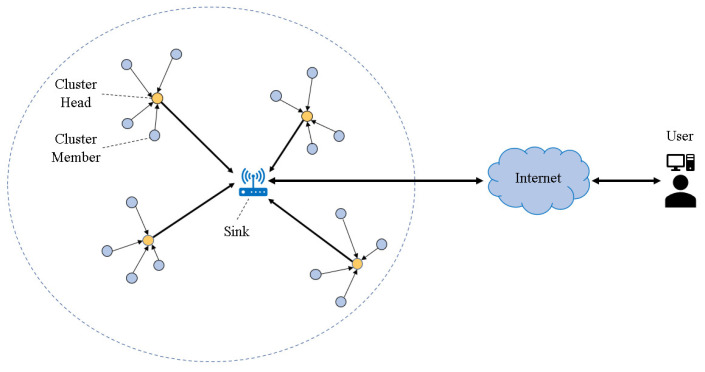
Clustered routing structure of WSN.

The selection of cluster head (CH) nodes in routing protocols is a key to efficient communication in WSN. The CH nodes undertake the tasks of information collection, data fusion and data transmission in the cluster, and their energy consumption is faster than other nodes. By designing an effective clustering protocol, each sensor node can use the limited energy more reasonably. The clustering routing protocol is divided into four processes: cluster head election, cluster formation, data fusion, and data transmission. It divides the entire network into multiple clusters. In each cluster, select one node as the cluster head (CH) node and the other nodes as the cluster member (CM) nodes. The CM nodes communicate with the CH node in each cluster and forward the data to the CH node. The CH nodes integrate the data and send it to the sink node, and then the sink node transmits the data to the network for communication management between users [5], as shown in Figure 1. In addition, the performance of routing clustering protocols in homogeneous networks is not good in heterogeneous networks, so the research based on clustering routing protocols is one of the research hotspots in heterogeneous networks at present.

Using the metaheuristic algorithm to design the cluster routing protocol has always been a popular direction of industry research [6]. The metaheuristic algorithm is a powerful tool to solve complex optimization problems [7]. It can obtain the best approximate solution for more complex NP-hard problems in polynomial time [8]. As the research on bionics becomes more and more mature, metaheuristic algorithms are proposed one after another; for example, the particle swarm optimization algorithm (PSO) [9,10], genetic algorithm (GA) [11,12], bat algorithm (BA) [13], seagull optimization algorithm (SOA) [14], and the grey wolf optimizer (GWO) [15], etc. The formulas for individual movements of many metaheuristic algorithms are based on operations such as addition, subtraction, multiplication, and division. The mathematical model of the algorithm is not closely related to the essence of things, and there is no specific scientific theory support. In order to come up with a metaheuristic algorithm with good performance and a close connection between the mathematical model and the essence of things, we searched for formula derivation about the growth principle of bamboo forest in biology. Based on the differential model of the bamboo growth and the Gaussian mixture models [16], this study proposes a new metaheuristic optimization algorithm named the bamboo forest growth optimizer (BFGO) and demonstrates the effectiveness of the algorithm’s optimization ability is proved on the CEC test sets and engineering optimization problems.

In addition, for the problem of energy consumption in WSN, based on the BFGO algorithm, this paper proposes an energy-efficient clustering mechanism of protocol (BFGO-C) for two-level heterogeneous WSN is proposed. The following are the characteristics of this study:

- A small number of heterogeneous nodes in WSN can usually be used to improve network life and stability, and different types of nodes have different initial energy and consumption rates. In the two-level heterogeneous WSN studied, only energy heterogeneity is considered, and the sensor nodes are divided into advanced nodes and normal nodes;
- The encoding method of the BFGO algorithm is redesigned in this paper. Each individual in the algorithm represents a set of cluster heads;
- The fitness function is improved for BFGO-C, which first considers the relationship between remaining energy and node energy, and also considers the separation of inter-cluster and the compactness of intra-cluster. The purpose of both is to reduce energy consumption and shorten the transmission distance of node communication;
- In experimental, using the four indicators of the lifetime of network, the lifetime of network until the first node dies, remaining energy, and data transmission volume to analysis, and it uses the entropy weight method [17] to conduct a comprehensive analysis.

The remaining structure of the paper is as follows. Section 2 reviews related work. Section 3 presents the BFGO algorithm. Section 4 analyzes the proposed network clustering mechanism of routing protocol (BFGO-C). Section 5 tests the performance of the BFGO algorithm. Section 6 simulates and analyzes the protocol in heterogeneous WSN. In Section 7, the conclusions are given.

## 2. Related Work

**Figure 2:**
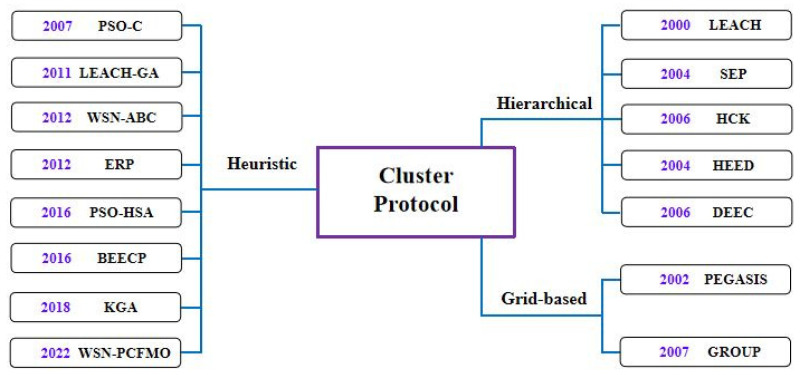
Related clustering protocols for WSN.

As the key to the clustering mechanism of routing protocol in WSN, many clustering techniques are applied to the clustering mechanism of WSN. The clustering schemes are divided into three categories: hierarchical clustering algorithm, heuristic clustering algorithm, and grid-based clustering algorithm. The related clustering protocol for WSN is shown in Figure 2.

### 2.1. Hierarchical Clustering Algorithm

Low energy adaptive clustering architecture (LEACH) [18], as the earliest network clustering routing protocol, randomly selects CH nodes in the cycle process and distributes the energy load evenly, effectively reducing network power consumption. Still, due to the randomness of cluster head selection, low-energy nodes can easily be selected as CH nodes and die, shortening the life of the network. Later, there were many improved versions of the LEACH protocol, such as mobile-LEACH [19], LEACH-balanced, LEACH-C [20], and other protocols. The stable election protocol (SEP) [21] is also improved based on the LEACH protocol, giving more energy to advanced nodes, effectively using heterogeneous networks. The cluster head selection of a hybrid, energy-efficient, distributed clustering approach (HEED) [22] focuses on the remaining energy and the energy consumed in the cluster, which improves energy utilization. Nodes in intelligent hierarchical cluster-based routing (HCR) [23] are self-organized into clusters, also improved using an agent-based architecture, resulting in energy-efficient hierarchical clusters. The distributed energy-efficient clustering algorithm (DEEC) [24] provides a different algorithm for estimating network lifetime and retains the distributed nature of the HEED protocol. As the most popular clustering algorithm, the LEACH protocol has been improved and applied by many subsequent algorithms.

### 2.2. Heuristic Clustering Algorithm

The heuristic clustering algorithm is a combination of a clustering algorithm and an intelligent algorithm based on biological principles. Due to the complexity of the problem and the high computational costs, people pay more attention to adding heuristic optimization algorithms to clustering algorithms to achieve more efficient results. PSO-clustering (PSO-C) is the earliest research that applies intelligent optimization algorithms to cluster routing protocols. It selects CH nodes by minimizing the distance between CHS and other nodes in the cluster [25]. In 2011, the adaptive probabilistic prediction clustering protocol, called LEACH-GA, was introduced, effectively extending the lifetime of the LEACH protocol [26]. In 2012, an artificial bee colony algorithm was used to cluster nodes in WSN, and energy was used as an indicator to design the fitness function [27]. In 2012, an evolutionary algorithm is used in routing clustering protocol (ERP), and a fitness function based on separation degree and cohesion degree is proposed [28]. In 2016, the PSO-HSA was improved based on PSO-C, and CH nodes were selected by combining the harmony search algorithm and PSO algorithm, which balanced the local constraints of the two algorithms and extended the life of the network [29]. In 2016, the biogeography optimization-based energy-efficient clustering protocol (BEECP) used the binary version of the biogeography optimization algorithm for clustering and optimized it with discrete coding, making the routing protocol more efficient to extend the life cycle [30]. In 2018, a protocol merged the GA algorithm with the K-means clustering algorithm (KGA) for cluster head selection in network [31]. In 2022, parallel fish migration optimization with compact technology based on the memory principle (PCMFO-Memory) proposed the idea of memory reduction, saving the best set of CH nodes in each round and applying it to the next round, which improved the efficiency of the clustering algorithm [32].

### 2.3. Grid-Based Clustering Method

Grid-based clustering method includes power-efficient gathering algorithm (PEGASIS) [33] and GROUP [34] protocols. Unlike the multi-cluster structure of the LEACH protocol, PEGASIS uses signal strength to measure the distance between nodes. It assumes that every node can communicate with the sink node. It is twice as fast as the LEACH protocol to improve the lifetime of the network. The cluster network established by the GROUP protocol is dynamically random. The grid seed is selected first, then the grid seeds along the radius identify the CH nodes in the grid. Its selection operation is based on the remaining energy in the node.

## 3. Proposed Bamboo Forest Growth Optimization Algorithm

### 3.1. Inspiration from the Growth Principle of Bamboo Forest

Bamboo is an herbaceous plant that grows explosively to the height of a tree. This rapid growth occurs during its shoot stage. As the “bamboo law” says, bamboo grows only 3 cm in four years and then grows at a rate of 30 cm per day from the fifth year onwards, reaching 15 m in just six weeks [35]. The bamboo extends its roots hundreds of square meters in the soil, and a short period of rapid growth occurs during the bamboo shoot. Therefore, the growth of bamboo forest can be divided into two stages: (a) the underground expansion of the bamboo whip; (b) the shoot growth of the bamboo.

In addition, a bamboo forest is composed of multiple bamboo whips, and the bamboos belonging to one bamboo whip are a group. The bamboo whip undergoes cell division and differentiation by absorbing nutrients from the soil to store energy. Some shoots emanating from the bamboo whip become vigorous bamboo shoots that burst from the ground, while others grow laterally and develop into new bamboo whips.

The two stages of bamboo forest growth can respectively correspond to the global exploration and local exploitation when the metaheuristic algorithm searches for solutions. So combined with the differential equation of bamboo forest growth, a bamboo forest growth optimizer (BFGO) algorithm can be constructed.

### 3.2. Mathematical Model

#### 3.2.1. Underground Extension of the Bamboo Whip

**Figure 3:**
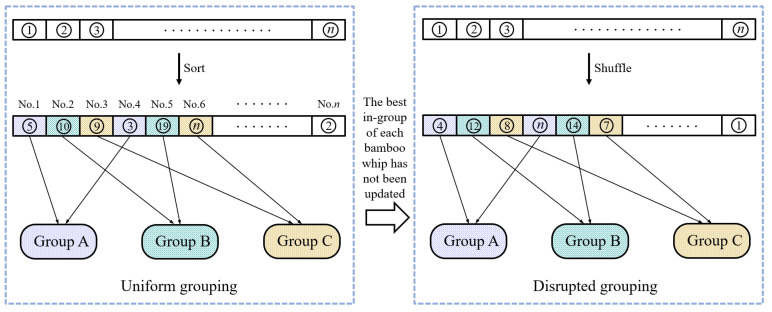
The idea of dynamic grouping.

Based on the characteristics of the bamboo whip, the idea of grouping is added to the algorithm, and individuals are dynamically grouped in the optimization process of the algorithm. Dynamic grouping is the dynamic scheduling of uniform grouping and disrupted grouping, and the fitness of individuals is used as the criterion for grouping. Among them, ’fitness’ refers to the performance of an individual to survive in the population. The description of the idea of dynamic grouping is shown in Figure 3.

The direction of the underground bamboo whip expansion depends on three factors: the directives for the group cognitive items, bamboo whip memory, and bamboo forest center, which means that the global optimum, the intra-group optimum, and the location of the central solution simultaneously affect the solution search direction. The formula for the direction of expansion is shown in Equations (1)–(3).

(1) $$cos\alpha =\frac{\vec{X_{t}}\cdot \vec{X_{G}}}{|\vec{X_{t}}\left|\times \right|\vec{X_{G}}|}$$

(2) $$cos\beta =\frac{\vec{X_{t}}\cdot \vec{X_{P(k)}}}{|\vec{X_{t}}\left|\times \right|\vec{X_{P(k)}}|}$$

(3) $$cos\gamma =\frac{\vec{X_{t}}\cdot \vec{C(k)}}{|\vec{X_{t}}\left|\times \right|\vec{C(k)}|},$$

where $\vec{X_{t}}$ is the position of the current solution, and $\vec{X_{G}}$ is the position of the globally optimal individual. $\vec{X_{P(k)}}$ and $\vec{C(k)}$ are the intra-group optimal solution and the central solution on the *k*-th bamboo whip, respectively. α, β and γ represent the extension direction of the current individual on $\vec{X_{G}}$, $\vec{X_{P(k)}}$ and $\vec{C(k)}$, respectively.

**Figure 4:**
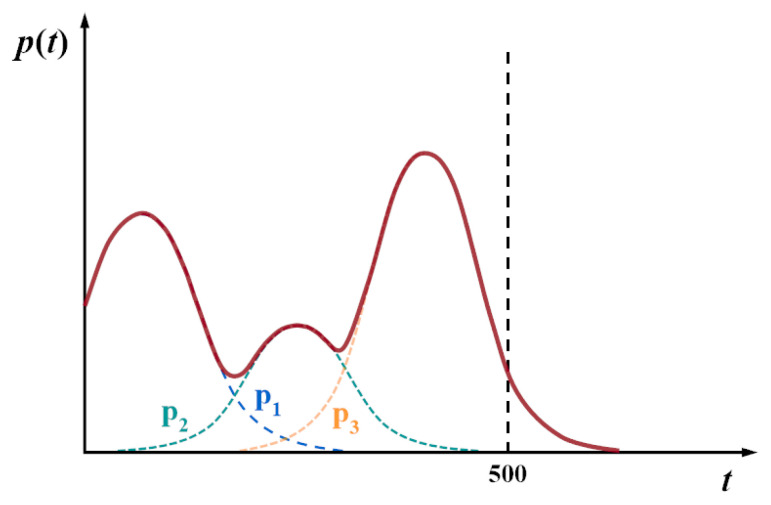
Gaussian mixture models for individual trend probabilities.

The formula for updating the solution at this stage is shown in Equation (4).

(4) $$X_{t+1}=\left\{\begin{array}{c} X_{G}+Q\times \left(c_{1}\times X_{G}-X_{t}\right)\times cos\alpha ,\;if\;R=P_{1}\left(t\right)\\ X_{P(k)}+Q\times \left(c_{1}\times X_{P(k)}-X_{t}\right)\times cos\beta ,\;if\;R=P_{2}\left(t\right)\\ C\left(k\right)+Q\times \left(c_{1}\times C\left(k\right)-X_{t}\right)\times cos\gamma ,\;if\;R=P_{3}\left(t\right) \end{array}\right.$$

(5) $$Q=2-\frac{t}{T},$$

where *Q* is a decreasing parameter, which decreases from 2 to 1 with the iteration of the algorithm, and can influence the development process of the algorithm to a certain extent; $c_{1}$ is a random number between 0 and 2. *t* represents the current iteration number, and *T* is the maximum iteration number; where *R* represents the probability of the moving direction of the individual and always takes the maximum probability of the three directions. $P_{1}\left(t\right)$, $P_{2}\left(t\right)$, and $P_{3}\left(t\right)$ are the probabilities of the moving trend of the solution simulated by the Gaussian mixture model, as shown in Figure 4. The dotted line parts represent the values of the three orientation probabilities, respectively, and the solid line parts represent the value of *R*.

The Gaussian mixture model moves individuals to a globally optimal solution in the early iteration process. As the number of iterations increases, the algorithm is more likely to fall into local optimization. To avoid the algorithm getting stuck in local optima, increase the probability of individuals tending towards a central solution. In this way, the distribution of solutions in the iterative process is more diverse, and the capacity of the algorithm to find the optimal solution is enhanced.

#### 3.2.2. Shoot Growth of the Bamboo

Combined with the stochastic process of the growth model proposed by Sloboda [36], different growth environments and random factors lead to different cumulative growth of each bamboo at time *t*. The cumulative growth of the shooting stage is shown in Equation (6).

(6) $$q\left(t\right)=X_{G}\times e^{d}\times e^{\frac{b}{\psi \times t^{\psi }}}.$$

**Figure 5:**
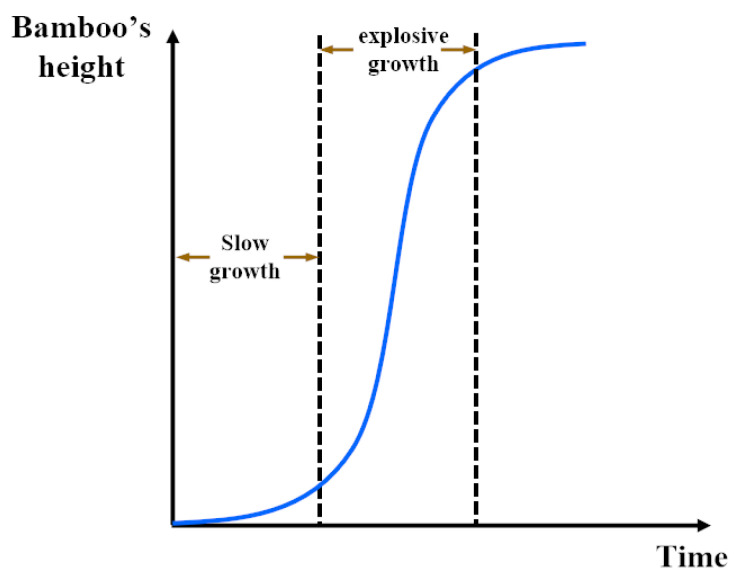
Growth trend of bamboo shoots.

The shape of the bamboo population incremental growth model is shown in Figure 5, including two stages: slow growth and explosive growth [37], where $X_{G}$ represents the maximum height of bamboo under a particular growth environment, *b* and ψ are the shape parameters of the model.

Given the bamboo accumulation at a specific time *t*, the change over time can be calculated, as shown in Equation (7).

(7) $$△H=\frac{q(t)-q(t-1)}{X_{G}-C\left(k\right)+1},$$

where △H represents the change in the cumulative amount of two iterations per unit distance, the denominator represents the distance from the optimal individual of the population to the center position, and q(t) represents the total cumulative amount of bamboo growth within the *t*-th generation.

The renewal of individuals in this phase is shown in Equations (8) and (9).

(8) $$X_{temp}=\left\{\begin{array}{c} X_{t}+X_{D}\times △H,rand<0.5\\ X_{t}-X_{D}\times △H,else \end{array}\right.$$

(9) $$X_{D}=1-|\frac{X_{t}-C\left(k\right)}{X_{G}-C\left(k\right)+1},|$$

where $X_{D}$ represents the ratio of the distance from the current individual to the optimal individual and the distance from the current individual to the central individual. Increases with the number of iterations, and the cumulative amount shows a trend of rapid growth in the early stage and slow growth in the late stage or even unchanged. Therefore, these two parameters will affect the breadth of algorithm exploration. In the stage of rapid accumulation growth, the algorithm is explored more widely, while in the stage of slow growth, the algorithm gradually reaches the convergence state.

In Equation (8), ‘+’ represents being away from the current individual, ‘−’ represents being close to the current individual. The smaller the value of $X_{D}$, the smaller the difference between the current individual and the optimal individual, and then search near the current individual. On the contrary, it is far away from the current individual to find a better solution.

## 4. Proposed Energy-Efficient Clustering Mechanism of Routing Protocol Based on BFGO Algorithm (BFGO-C)

**Figure 6:**
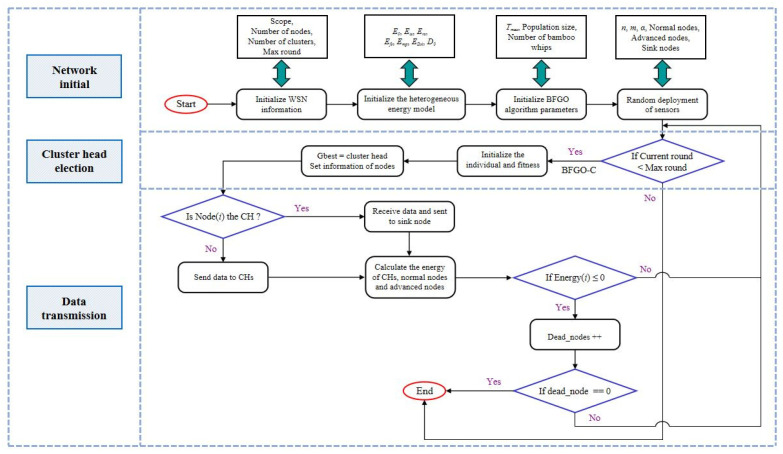
The working process of the protocol based on BFGO-C in HWSN.

This study presents an energy-efficient clustering mechanism protocol based on the BFGO algorithm. The goal of this work is to rationalize node distribution and minimize the energy consumption of the network. Then, the optimal set of cluster heads is selected to undertake the tasks of data collection, fusion, and transmitting in WSN. The protocol of the BFGO-C consists of a system model, an energy consumption model, and a cluster head election model. The system model introduces the assumptions for the HWSN simulation [30]. The energy consumption model calculates the energy consumption of sensor nodes. The cluster head election model describes the detailed processing of the cluster head selection by the BFGO-C. The working process of the protocol based on BFGO-C is shown in Figure 6. The pseudo-code Algorithm 1 of BFGO-C implemented in the routing protocol is as follows.
**Algorithm 1** BFGO-C Implemented in Routing Protocol.1:**//Initialization**Initialize node information, the energy of nodes in HWSN, the deployment of the nodes in WSN, and parameters in BFGO-C. Initialize the maximum number of running rounds ($R_{m}$), the current number of running rounds ($R_{c}$), the set of cluster heads ($CH_{s}$), the numbers of nodes (*N*), the number of dead advanced nodes ($N_{a}$), the number of dead normal nodes ($N_{n}$), and survival state of the network ($N_{s}$)2:**while** ($R_{c}$ ≤ $R_{m}$) **do**3:    **if** $N_{s}$ == false **then**4:        end the network5:    **end if**6:    **for** j = 1; j ≤ N; j + + **do**7:        **if** energy ≤ 0 **then**8:             $N_{a}$++;9:             $N_{n}$++;10:        **end if**11:    **end for**12:    **//Phase 1: Cluster head election**Initialize the nummber of current iteration (t), the nummber of max iteration (T), the individual ($X_{t}$) and calculate the individual objective function value ($f_{t}$) by Equations (15) and (16)13:    **while** (*t* ≤ *T*) **do**14:        update $X_{t}$ using Equations (1)–(5), sort and update $X_{G}$, $X_{P(k)}$, and C(k)15:        update $X_{t}$ using Equations (6)–(9), sort and update $X_{G}$, $X_{P(k)}$, and C(k)16:        **for** j = 1; j ≤ k; j + + **do**17:           **if** $X_{P(k)}$ not updated **then**18:               count++;19:           **end if**20:        **end for**21:        **if** count == k **then**22:           Do dynamic updates23:        **end if**24:        $CH_{s}$=$X_{G}$;25:    **end while**26:    **//Phase 2: Elected cluster head for data transmission**27:    Calculate the number of cluster heads ($N_{ch}$)28:    **for** i = 1; j ≤ N; j + + **do**29:        **if** Node(i) == cluster head; **then**30:           Data transmission31:           Calculate consumed energy, remaining energy, and transfer volume using Equations (11)–(16)32:        **else** Send data to cluster head33:        **end if**34:    **end for**35:**end while**

### 4.1. System Model

The Heterogeneous network is a two-level network [38], and the nodes include advanced nodes and normal nodes. It is assumed that *n* is the number of nodes, *m* is the proportion of advanced to all nodes, and its energy is α times higher than that of normal nodes. Let the initial energy of normal nodes be $E_{0}$, then the initial energy of advanced nodes is $E_{0}\times \left(1+\alpha \right)$. Then the total node energy of the entire HWSN is:

(10) $$E_{total}=n\times \left(1-m\right)\times E_{0}+n\times m\times \left(1+\alpha \right)\times E_{0}=n\times E_{0}\times \left(1+\alpha \times m\right).$$

There is a WSN randomly and uniformly deployed by *N* nodes to collect data [39]. The following assumptions are made within this simulation environment:

(a) Nodes are static. The base station node is unique and Located centrally in the area of the network;
(b) All nodes have unique identification numbers;
(c) The CH node is responsible for data fusion and transmits the fused data to the base station;
(d) The energy of sensor nodes is limited. Once they die, they can no longer participate in the network;
(e) Nodes can calculate and store data, and obtain their residual energy and distance from other nodes;
(f) The sink node uses a fixed power supply and does not die;
(g) Only the energy heterogeneity of nodes is considered, and other heterogeneity characteristics are not considered.

### 4.2. Energy Consumption Model

**Figure 7:**
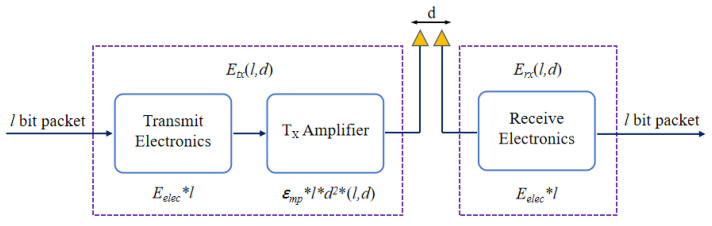
Energy consumption model.

The energy consumption of the network mainly comes from the wireless communication between nodes. According to the first-order radio model, this study uses the energy loss formula to calculate the energy consumption of nodes. The model diagram is shown in Figure 7, The sensor node consists of a transmitter and a receiver [40]. The left module is the transmitter of the sensor node, and the right module is the receiver. *l* is the length (in bits) of the information received or transmitted by the sensor node. *d* is the distance between the transmitter and the receiver.

As shown in Figure 7, the data are transmitted from the transmitting electronics to the amplifier. IThey are transmitted to the receive electronics at a distance of *d* meters through wireless communication [41]. When sending data, the energy consumption produced by the node is the sum of the energy consumption produced by the transmitting electrons and the amplifier circuit [42]. The calculation of transmitting volume of routing protocol based on BFGO-C is shown in Equation (11).

(11) $$E_{tx}\left(l,d\right)=\left\{\begin{array}{c} E_{elec}\times l+\epsilon_{fs}\times l\times d^{2},d<d_{0}\\ E_{elec}\times l+\epsilon_{mp}\times l\times d^{4},\mathrm{else} \end{array}\right.$$

where $E_{elec}$ represents the power used by the transmitter and receiver, and $\epsilon_{fs}$ and $\epsilon_{mp}$ are the power amplifier coefficients under the free space and multipath fading models, respectively. $d_{0}$ represents the distance threshold from the transmitter to the receiver, as shown in Equation (12).

(12) $$d_{0}=\sqrt[{2}]{\frac{\epsilon_{fs}}{\epsilon_{mp}}}.$$

If $d>d_{0}$, it is a multipath fading model; conversely, it is a free space model. The received data energy consumption $E_{rx}$ is shown in Equation (13).

(13) $$E_{rx}\left(l\right)=E_{elec}\times l.$$

The energy consumption of data fusion is shown in Equation (14).

(14) $$E_{m}\left(l\right)=E_{DA}\times l\times \left(1+n\right),$$

where $E_{DA}$ is the unit energy consumption of data fusion.

### 4.3. Cluster Head Election Model

#### 4.3.1. Encoding Method

The correspondence between the concepts of the clustering mechanism and the BFGO algorithm is shown in the Table 1. As shown in the first row of Table 1, a set of CH nodes in the clustering mechanism is equivalent to a bamboo individual in the BFGO algorithm. In the second row, the number of CH nodes is equivalent to the dimension of the individual, and so on.

#### 4.3.2. Fitness Function

From the description of the coding method, It can be known that the fitness function determines the quality of the set of CH nodes by evaluating the individual [43]. The fitness function in this paper is designed according to three factors: the compactness within the cluster, the separation between the clusters, and the relationship between the initial energy and the remaining energy. Among them, the relationship between the initial energy and the remaining energy is an essential factor, because the difference between HWSN and WSN lies in the setting of the initial energy of the sensor node. In advanced nodes, the energy factor accounts for a more significant proportion [44], So that the high-energy nodes as the CH nodes can effectively extend the lifetime of the network. At the same time, the low-energy CH nodes lead to a faster node death rate. The fitness function is shown in Equation (15).

(15) $$Fitness=\sum_{C=1}^{CHs}\frac{E_{0}}{{\left(E_{r}\right)}^{p}}+\sum_{i=1}^{CHs}\sum_{j=1}^{N_{i}}dis\left(S_{j},CH_{i}\right)+\sum_{i=1}^{CHs}\sum_{j=1}^{CHs}dis\left(CH_{i},CH_{j}\right)$$

(16) $$dis\left(X,Y\right)=\sqrt[{2}]{\sum_{i=1}^{n}{\left(x_{i}-y_{i}\right)}^{2}},$$

where $E_{0}$ is the initial energy of the node, $E_{r}$ is the current remaining energy of node *i*, and *p* is the weight coefficient. The ratio of the residual energy of the node to the initial energy can reflect the current residual energy of the CH node. $N_{i}$ represents the number of nodes in the *i*-th cluster, and $S_{j}$ is the non-cluster head node in the *j*-th cluster, $dis(CH_{i},CH_{j})$ represents the distance between any two cluster heads. The Euclidean distance is used to calculate both compactness and separation, as shown in Equation (16).

## 5. Performance Test of BFGO Algorithm

The CEC test suite is a set of functions commonly used for testing and evaluating the performance of the metaheuristic algorithm, including unimodal functions, simple multimodal functions, hybrid functions, and composite functions. The tests of multi-type functions can show the performance of the algorithm more comprehensively. To test the optimization performance of the BFGO algorithm, tests and analysis are conducted in the CEC2013 benchmark function set [45], CEC2017 benchmark function set [46], and three engineering optimization problems [47]. Table 2 summarizes the relevant parameters, ’own’ represents the parameters set by the algorithm itself, and ’unified’ is the public parameter in the experiment.

### 5.1. Test of CEC2013 Benchmark Function

**Figure 8:**
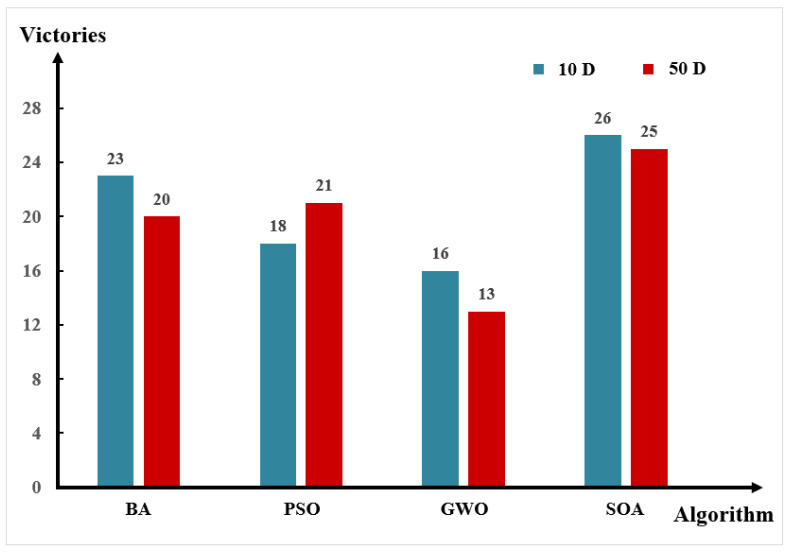
Comparison of BFGO algorithm getting better times in CEC2013 test.

The experimental results of this part are shown in the Appendix A, Figure 8 shows the number of functions the BFGO algorithm outperforms in other algorithms in 10-dimensional (10D) and 50-dimensional (50D) spaces.

From Table A1, among the 28 functions of the CEC2013 test set, the BFGO algorithm is superior to the BA algorithm, PSO algorithm, GWO algorithm, and SOA algorithm in 23, 18, 16, and 26 functions. It can be seen from Table A2 that the BFGO algorithm is superior to the BA algorithm, PSO algorithm, GWO algorithm, and SOA algorithm in 20, 21, 13, and 25 functions, respectively. It can be seen that the BFGO algorithm has advantages over other algorithms in both low and high dimensions, especially in low dimensional space, showing strong competitiveness.

The scalability of the algorithm in CEC2013 can be analyzed in Figure 8. The performance of the BFGO algorithm decreases with the increase of dimension. However, it is still better than the other algorithms in most functions.

### 5.2. Test of CEC2017 Benchmark Function

**Figure 9:**
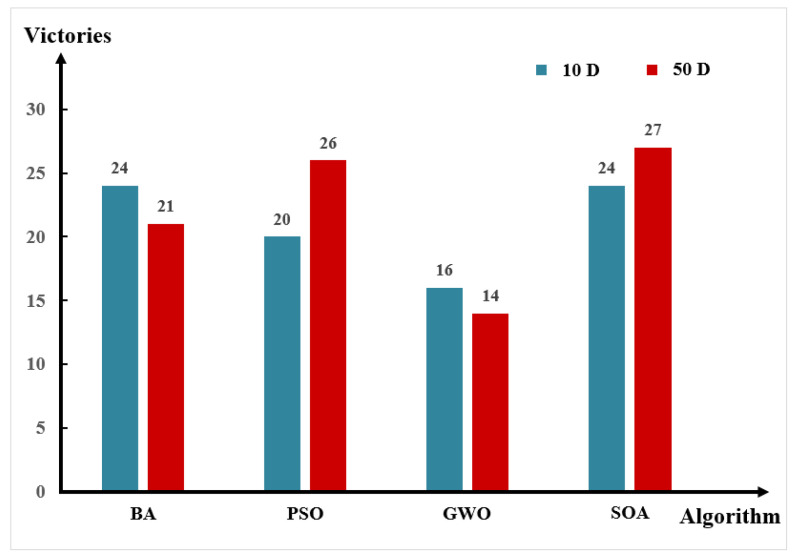
Comparison of BFGO algorithm getting better times in CEC2017 test.

The experimental results of this part are shown in the Appendix A, Figure 9 shows the number of functions the BFGO algorithm outperforms in other algorithms in 10D and 50D space.

It can be seen from Table A3 that for the 30 functions of CEC2017, the overall performance of BFGO is better than other algorithms on 10D. The BFGO algorithm is superior to the BA algorithm, PSO algorithm, GWO algorithm, and SOA algorithm in 24, 20, 16, and 24 functions. It can be seen from Table A4 that the BFGO algorithm also has good performance on 50-D. The BFGO algorithm is superior to the BA algorithm, PSO algorithm, GWO algorithm, and SOA algorithm in 21, 26, 14, and 27 functions.

The scalability of the algorithm in CEC2017 can be analyzed in Figure 9. The performance of the BFGO algorithm decreases slightly with the increase of dimension. However, it is still better than the other algorithms in most functions.

According to the test results of five algorithms in the CEC2013 and CEC2017, the BFGO algorithm is significantly better than the BA, PSO, and SOA algorithms in both low-dimensional and high-dimensional space, and its performance is similar to the GWO algorithm, only slightly lower than the GWO algorithm in high-dimensional space. To sum up, the BFGO algorithm has good optimization performance and strong competitiveness.

### 5.3. Test of Engineering Optimization

Three classical engineering optimization problems are used to test the performance of the BFGO algorithm in finding appropriate parameters to optimize the solution of practical problems, including compression spring design, welded beam design, and speed reducer design [48,49]. The three issues are optimized with parameters 3, 4, and 7 to minimize cost dissipation.

Table 3, Table 4 and Table 5 compare the results of the BFGO algorithm with the BA, GWO, PSO, and SOA algorithms tested on the three engineering design problems 30 times, respectively. The table shows the optimal parameters, the mean (Mean), the standard deviation (Std), and the minimum (Min) values for the 30 tests. Where underline represents the same optimum and bold represents the optimum.

In the test of compression spring design, the BGFO algorithm obtains the same optimal value as the GWO and SOA algorithms in the mean and minimum values. The standard deviation is slight, indicating that the algorithm is stable. In the test of welded beam design, the mean value of 30 tests obtained by the BFGO algorithm ranks second, second only to the GWO algorithm, and other algorithms are still far behind the BFGO algorithm. Similarly, in the speed reducer design problem, the mean value of the BFGO algorithm in the 30 tests is the first, the minimum value is tied for the first best with the GWO algorithm, and the standard deviation is still tiny, indicating that the algorithm is relatively stable.

Therefore, compared with other algorithms, the BFGO algorithm also has powerful optimization ability for engineering optimization problems.

## 6. Simulation and Analysis of Protocol Based on BFGO-C in HWSN

**Figure 10:**
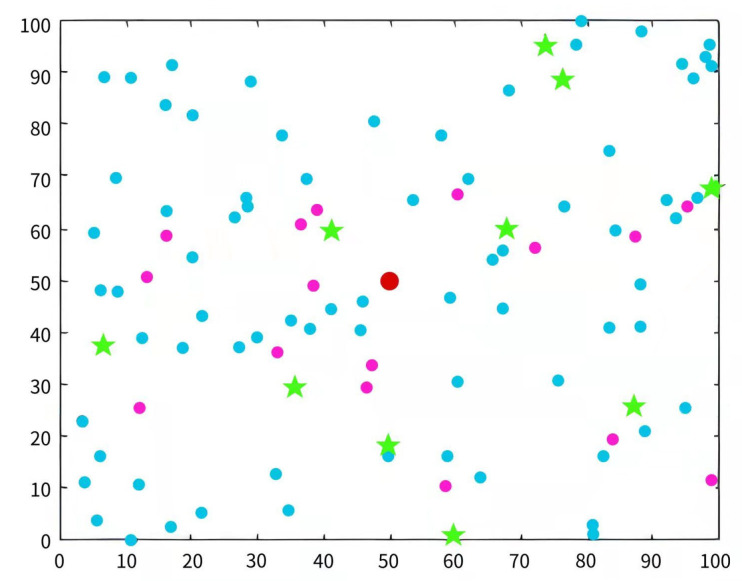
Initial node distribution in the simulation environment.

**Figure 11:**
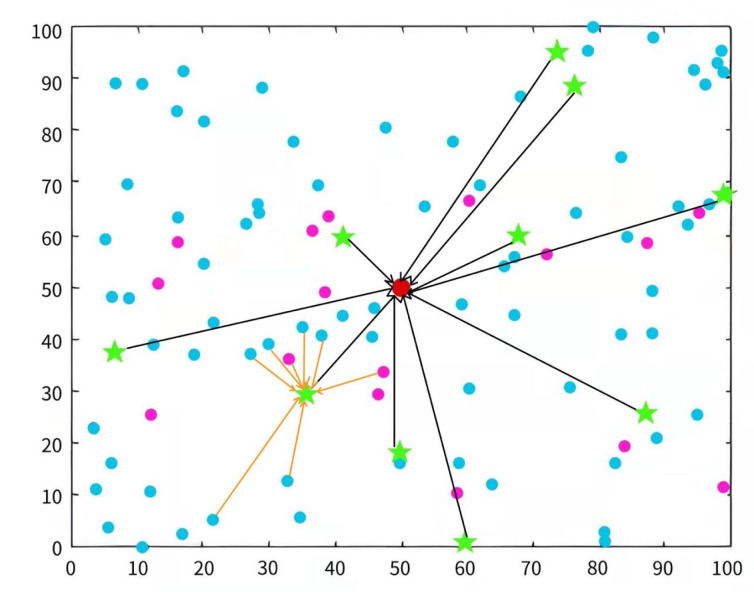
Initial node interaction in the simulation environment.

This study simulates the protocol based on the BFGO-C and compares the results with the SEP, HCR, and ERP clustering protocols. Table 6 describes the parameters in the simulation experiments. In this paper, four indicators are used as measures to analyze the experimental results: the life of the network, the life of the network until the first node dies, energy consumption, and the data transmission volume. Figure 10 shows the assignment of initial CH nodes in the 100 × 100 area, where the red dot in the middle is the sink node, the blue dot is the normal node, the purple dot is the advanced node, and the green pentagram is the initial CH node [50]. Figure 11 is the initial node interaction diagram. The black arrows indicate that the CH nodes transmit data to the sink node, and the orange arrows indicate that the nodes in the cluster transmit data to the CH nodes [51].

### 6.1. Comparison of the Lifetime of Network

**Figure 12:**
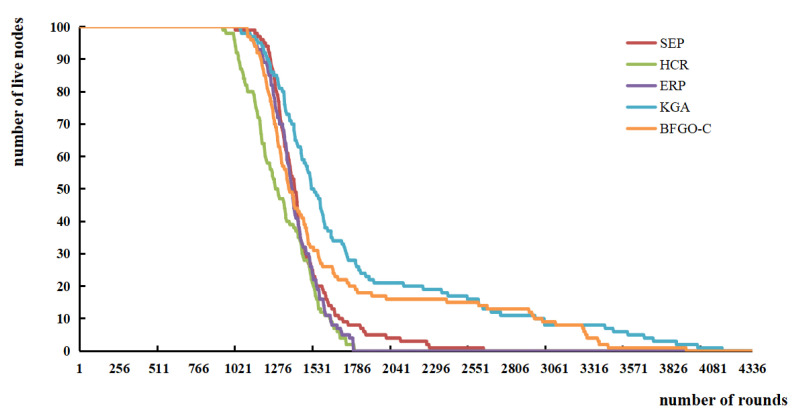
Variation of the number of live nodes with the number of rounds.

The number of rounds in which the last node dies represents the life of the network. Figure 12 shows the changes in the surviving nodes of the SEP protocol, the HCR protocol, the ERP protocol, and the protocol based on BFGO-C with the number of rounds.

The survival trend of the four clustering protocols SEP, HCR, ERP, and the protocol based on BFGO-C in Figure 12. As the network runs, the consume energy of nodes for data transmission and forwarding. After several operation rounds, some nodes run out of energy and become dead nodes. The graph shows that the nodes of the protocol based on BFGO-C die a little slower, and the network lives a little longer than all three other protocols.

**Figure 13:**
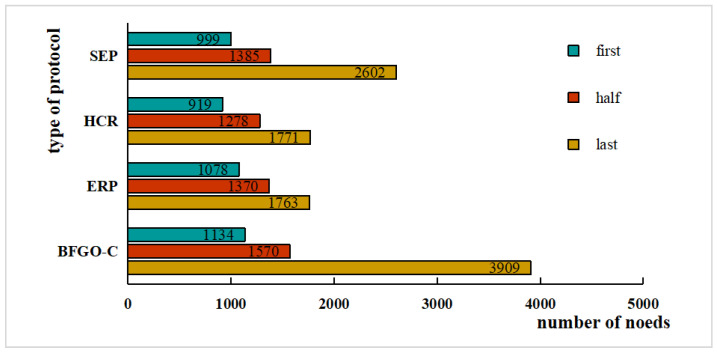
Number of surviving nodes for rounds when the first node dies, half of the nodes die, and the last node dies.

There are also application scenarios that pay more attention to the comprehensiveness of the node information. Once the first node in the network dies, the data are missing by default. Hence the paper records the number of rounds when the first node dies, half of the nodes die, and the last node dies, as shown in Figure 13.

In Figure 13, the growth cycles of the four cluster protocols SEP, HCR, ERP, and the protocol based on BFGO-C are 2602, 1771, 1763, and 3909 rounds, respectively. The protocol based on BFGO-C has the longest lifetime of the network. In SEP protocol, the network ran 999 rounds when the first node died and 1385 rounds when half of the nodes died. The first node of the HCR and ERP protocol died in rounds 919 and 1078, and half of the nodes died in rounds 1771 and 1763. The number of rounds when the first node of the protocol based on BFGO-C died was 1134, and the number of rounds when the intermediate node died was 1570.

It can be shown that the protocol based on BFGO-C has a higher number of rounds at the death of the first node and a higher number of rounds at the end of half of the nodes than the other three protocols.

### 6.2. Comparison of Remaining Energy

**Figure 14:**
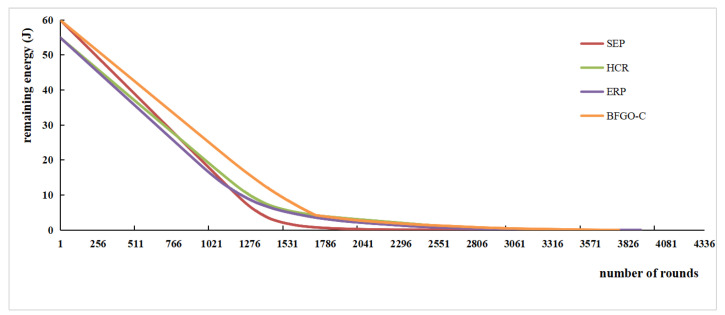
Variation of remaining energy with the number of rounds.

**Figure 15:**
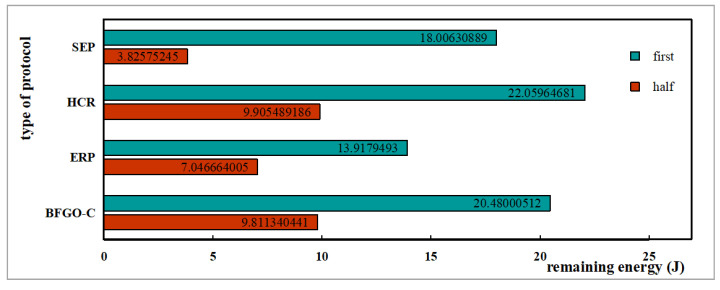
Remaining energy for key rounds.

The energy consumption during network operation reflects the performance of the network. More remaining energy indicates less energy consumption and better network performance. The variation of remaining energy with the number of rounds for the protocols SEP, HCR, ERP, and the protocol based on BFGO-C running in the network is shown in Figure 14, and the remaining energy for the rounds when the first node dies and half of the nodes die is shown in Figure 15.

The trend in energy consumption from Figure 14 shows that the protocol based on BFGO-C has more remaining energy than SEP, HCR, and ERP protocols. At the death of the first node in the network, the HCR protocol has the most residual energy and consumes the least, and the protocol based on BFGO-C is second. At the death of half of the nodes, the protocol based on BFGO-C and HCR protocol have similar residual energy, and both consume less energy than the SEP and ERP protocols. At the death of all nodes, the protocol based on BFGO-C consumes the least total energy than SEP, HCR, and ERP protocols. Protocol based on BFGO-C reduces network energy consumption to a certain extent.

### 6.3. Comparison of the Data Transmission Volume

**Figure 16:**
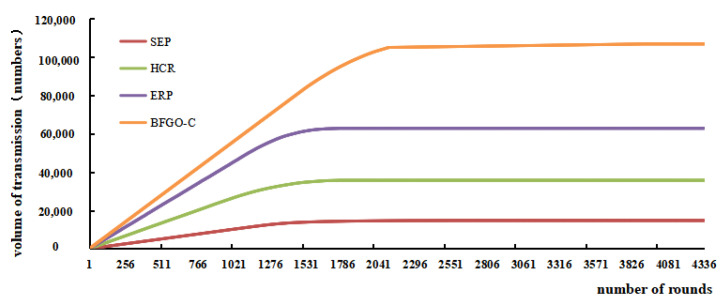
Variation of the volume of transmission with the number of rounds.

**Figure 17:**
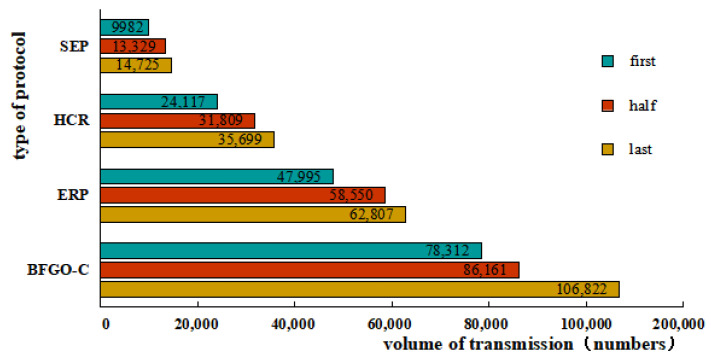
The volume of transmission for key rounds.

The surviving nodes in the network transmit a data packet to the CH nodes every round, and then the CH nodes transmit it to the base station, and the base station counts the number of data packets collected. The data transmission volume represents the total number of packets sent, it reflects the throughput of the network. The data transmission volume of the four protocols is shown in Figure 16, and Figure 17 also shows the data transmission volume when the first node dies, half of the nodes die and the last node dies.

The trend of the data transmission volume shows that the protocol based on BFGO-C has the fastest and highest transmission volume. With all the nodes dead, the number of packets stopped growing, and the SEP protocol, HCR protocol, ERP protocol, and protocol based on BFGO-C had 14,725, 35,699, 628,07, and 122,068 data transmissions, respectively, with a protocol based on BFGO-C eventually transmitting the most data. The protocol based on BFGO-C was consistently ahead of the other protocols regarding the number of packets transmitted in the early and middle stages of the network.

The protocol based on BFGO-C has achieved good performance in HWSN network clustering. Compared with the SEP, HCR and ERC protocols, it extends network life to a certain extent, reduces network energy consumption, and effectively improves network performance.

### 6.4. Comprehensive Evaluation Based on Entropy Weight Method

Information entropy can measure the discreteness of indicators, and the larger the discreteness, the more significant the impact of the index on a comprehensive evaluation. The entropy weight method determines the index’s weight in the comprehensive evaluation according to the variability of the information entropy reaction of the index.

**Figure 18:**
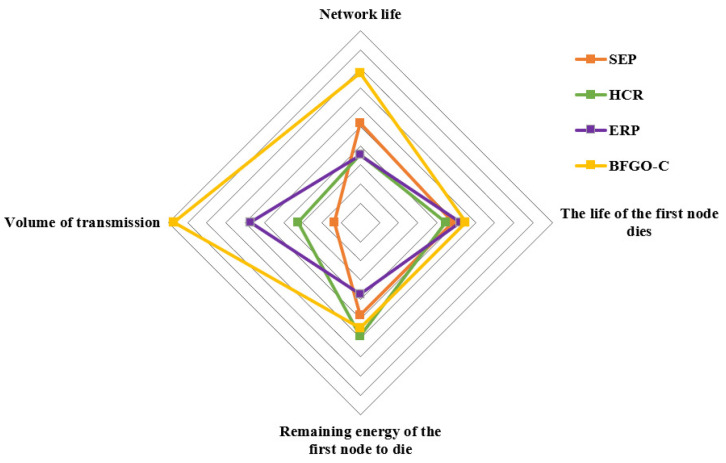
The radar chart of the comprehensive performance.

From the simulation results and analysis of the protocol based on BFGO-C in Section 5, the entropy weight method is used to comprehensively evaluate the four protocols in combination with four metrics: the lifetime of the network, the death time of the first node, remaining energy, and the volume of transmission. The radar chart of the comprehensive analysis is shown in Figure 18.

As can be seen from Figure 18, the comprehensive performance of the protocol based on BFGO-C covers the most extensive range, and it has outstanding performance in the two indicators of network life and volume of transmission, although it is slightly worse in the indicator of the remaining energy of the first node death. Compared with the HCR protocol, the energy of the protocol based on BFGO-C lasts longer in the network. It can be seen from the comprehensive evaluation that the protocol based on BFGO-C can effectively extend the network lifetime by saving energy.

## 7. Conclusions

This paper proposes an energy-efficient clustering mechanism of routing protocol for heterogeneous WSN based on the BFGO algorithm. Its core concept is to use the optimization ability of the BFGO algorithm to conduct cluster head selection, find the optimal set of CH nodes, guarantee the rationality of the cluster allocation, and maximize the network performance. First, based on the growth characteristics of a bamboo forest, a bionic intelligent optimization algorithm is proposed for the optimization problem. The algorithm has been shown to be highly competitive in both low-dimensional and high-dimensional spaces for CEC2013 and CEC2017 test functions and engineering optimization problems. The fitness function is redesigned when the BFGO algorithm is applied to the clustering mechanism of the routing protocol in heterogeneous WSN. Not only are the intra-cluster compactness and inter-cluster separation of the clusters considered, but the ratio of the initial to remaining energy is also taken as an important measure.

This study compared the protocol based on BFGO-C with the SEP, HCR, and ERP protocols using four indicators in simulation experiments. The experimental results show that the algorithm can reduce the network energy consumption, extend the network life, and significantly improve the data transmission volume. Finally, to evaluate the clustering performance of these protocols comprehensively, the study used the entropy weight method to give weights and comprehensive analysis, and the results prove that the comprehensive performance of the protocol based on BFGO-C is greater than the other protocols.

This paper does not consider other types of node performance heterogeneity, such as computational or link heterogeneity. In the future, we will continue to investigate how to improve and optimize the cluster routing protocols in heterogeneous and complex environments.

## Figures and Tables

**Table 1:** The correspondence between the concepts of the clustering mechanism and the BFGO algorithm.

Clustering Mechanism	BFGO Algorithm
a set of CH nodes	a bamboo individual
the number of cluster heads	the dimensions of individual
the effectiveness of a set of CH nodes	the fitness of a bamboo individual
the optimal set of CH nodes	the global optimal individual

**Table 2:** Parameters of the relevant algorithm.

Algorithm	Parameter (Own)	Parameter (Unified)
BA [13]	$A_{i}$ = 0.6, *r* = 0.7, $A_{f}$ = 0.9, $R_{f}$ = 0.9, $Q_{min}$ = 0, $Q_{max}$ = 1	Runs = 30, Population = 100, iterations = 500, lb = −100, ub = 100, dimension = 10D/50D
PSO [9]	*c*_1_ = 2, $c_{2}$ = 2	
GWO [15]	none	
SOA [14]	none	
BFGO	bamboowhips = 5, sita = 2	

**Table 3:** Comparison of BFGO algorithm and other algorithms in optimizing compression spring design.

Algorithm	Optimize Variable			Mean	Std	Min
	d	D	N			
BA	0.050000	0.282000	2.000000	0.002824	3.3397 × 10${}^{-6}$	0.002820
PSO	0.052700	0.277500	4.136000	13,186.75	7.2227 × 10${}^{4}$	0.004731
GWO	0.050000	0.282000	2.000000	0.002820	1.5421 × 10${}^{-8}$	0.002820
SOA	0.050000	0.282000	2.000000	0.002820	1.1723 × 10${}^{-7}$	0.002820
BFGO	0.050000	0.282000	2.000000	0.002820	4.6049 × 10${}^{-8}$	0.002820

**Table 4:** Comparison of BFGO algorithm and other algorithms in optimizing welded beam design.

Algorithm	Optimize Variable				Mean	Std	Min
	$T_{s}$	$T_{h}$	R	L			
BA	0.196600	0.101700	10.193600	67.907400	3074.41	7.7449 × 10${}^{3}$	119.658
PSO	1.584700	20.84740	10.100800	104.54950	103,174.1	9.9926 × 10${}^{4}$	6158.07
GWO	0.192900	0.095300	10.000000	64.124600	**108.910**	5.688 × 10${}^{-3}$	108.902
SOA	0.192900	0.095200	10.000000	64.197300	5617.420	1.3710 × 10${}^{4}$	109.001
BFGO	0.192800	0.095400	10.000000	64.270600	112.824	4.5868	109.097

**Table 5:** Comparison of BFGO algorithm and other algorithms in optimizing speed reducer design.

Algorithm	Optimize Variables							Mean	Std	Min
	$x_{1}$	$x_{2}$	$x_{3}$	$x_{4}$	$x_{5}$	$x_{6}$	$x_{7}$			
BA	3.600000	0.800000	28.000000	7.300000	7.800000	3.900000	5.283700	201,614.63	1.592047	201,613.20
PSO	3.492600	0.792700	25.847700	7.691400	8.274700	3.841700	5.315900	540,799.84	108,075.4	369,151.63
GWO	3.600000	0.800000	28.000000	7.300000	7.800000	3.900000	5.284700	201,613.24	0.101490	201,613.19
SOA	3.600000	0.800000	28.000000	7.300000	7.800000	3.900000	5.284900	201,616.66	4.204730	201,613.20
BFGO	3.600000	0.800000	28.000000	7.300000	7.800000	3.900000	5.284700	**201,613.19**	0.181397	201,613.19

**Table 6:** Simulation parameters of protocol based on BFGO-C in HWSN.

Simulation Parameters	Value
Network area	100 × 100 m^2^
Number of nodes (*N*)	100
Base station position	(50,50)
Packet size (l)	4000 bits
Initial energy of node ($E_{0}$)	0.5 J
Advanced node scale (*m*)	0.1 or 0.2
Transmitter/Receiver electronics ($E_{elec}$)	50 n J/bit
Transmit amplifier (free space) $\epsilon_{fs}$	10 nJ/bit/m^2^
Transmit amplifier (multipath) $\epsilon_{mp}$	0.0013 nJ/bit/m^4^
Data aggregation energy cost $E_{DA}$	5 nJ/ bit
Number of optimized individuals	20
Number of iterations	20
The weight of the fitness function	4

## Data Availability

Not applicable.

## Appendix A. Test Results on CEC Test Set

**Table A1:** Comparison of BFGO algorithm and other algorithms on CEC2013 benchmark function under 10D.

$f_{x}$	BA		PSO		GWO		SOA		BFGO	
	Mean	Std	Mean	Std	Mean	Std	Mean	Std	Mean	Std
$f_{1}$	−1.399 × 10${}^{3}$	1.1848 × 10${}^{-1}$	−1.339 × 10${}^{3}$	2.3095 × 10${}^{2}$	−1.385 × 10${}^{3}$	4.7240 × 10${}^{1}$	−1.068 × 10${}^{3}$	1.9838 × 10${}^{2}$	−1.400 × 10${}^{3}$	3.1599 × 10${}^{2}$
$f_{2}$	1.7685 × 10${}^{5}$	1.3611 × 10${}^{5}$	6.5680 × 10${}^{5}$	1.7462 × 10${}^{6}$	1.0902 × 10${}^{6}$	9.4889 × 10${}^{5}$	2.1364 × 10${}^{6}$	1.7465 × 10${}^{6}$	2.9837 × 10${}^{5}$	2.4558 × 10${}^{5}$
$f_{3}$	2.1322 × 10${}^{7}$	3.9879 × 10${}^{7}$	7.7272 × 10${}^{8}$	1.2511 × 10${}^{9}$	4.3836 × 10${}^{7}$	9.7444 × 10${}^{7}$	6.4612 × 10${}^{8}$	6.1898 × 10${}^{8}$	2.7794 × 10${}^{8}$	4.4521 × 10${}^{8}$
$f_{4}$	1.5171 × 10${}^{4}$	7.0814 × 10${}^{3}$	7.9318 × 10${}^{2}$	7.9543 × 10${}^{3}$	7.1724 × 10${}^{3}$	3.8745 × 10${}^{3}$	6.1317 × 10${}^{3}$	2.2208 × 10${}^{3}$	2.1336 × 10${}^{3}$	1.7939 × 10${}^{3}$
$f_{5}$	−9.996 × 10${}^{2}$	6.7815 × 10${}^{-2}$	−9.759 × 10${}^{2}$	4.5922 × 10${}^{1}$	−9.778 × 10${}^{2}$	1.4966 × 10${}^{1}$	−8.781 × 10${}^{2}$	1.6622 × 10${}^{2}$	−9.999 × 10${}^{2}$	4.8037 × 10${}^{-2}$
$f_{6}$	−8.916 × 10${}^{2}$	1.9843 × 10${}^{1}$	−8.783 × 10${}^{2}$	2.6334 × 10${}^{1}$	−8.748 × 10${}^{2}$	2.5992 × 10${}^{1}$	−8.372 × 10${}^{2}$	4.0619 × 10${}^{1}$	−8.837 × 10${}^{2}$	2.7752 × 10${}^{1}$
$f_{7}$	−6.442 × 10${}^{2}$	1.0048 × 10${}^{2}$	−7.591 × 10${}^{2}$	2.4188 × 10${}^{1}$	−7.898 × 10${}^{2}$	8.1917	−7.655 × 10${}^{2}$	1.1585 × 10${}^{1}$	−7.592 × 10${}^{2}$	3.3546 × 10${}^{1}$
$f_{8}$	−6.795 × 10${}^{2}$	9.6019 × 10${}^{-2}$	−6.796 × 10${}^{2}$	9.4408 × 10${}^{-2}$	−6.796 × 10${}^{2}$	7.8637 × 10${}^{-2}$	−6.797 × 10${}^{2}$	7.8866 × 10${}^{-2}$	−6.797 × 10${}^{2}$	9.3192 × 10${}^{-2}$
$f_{9}$	−5.914 × 10${}^{2}$	1.7614 × 10	−5.946 × 10${}^{2}$	1.3188	−5.963 × 10${}^{2}$	1.3082	−5.934 × 10${}^{2}$	1.2878	−5.938 × 10${}^{2}$	1.3414
$f_{10}$	−4.990 × 10${}^{2}$	1.0014 × 10${}^{-1}$	−4.638 × 10${}^{2}$	3.9716 × 10${}^{1}$	−4.896 × 10${}^{2}$	1.0867 × 10${}^{1}$	−4.447 × 10${}^{2}$	3.4506 × 10${}^{1}$	−4.987 × 10${}^{2}$	7.5568 × 10${}^{-1}$
$f_{11}$	−3.194 × 10${}^{2}$	3.4114 × 10${}^{1}$	−3.801 × 10${}^{2}$	1.0161 × 10${}^{1}$	−3.890 × 10${}^{2}$	7.2581	−3.643 × 10${}^{2}$	1.2287 × 10${}^{1}$	−3.793 × 10${}^{2}$	1.1004 × 10${}^{1}$
$f_{12}$	−2.072 × 10${}^{2}$	3.8047 × 10${}^{1}$	−2.743 × 10${}^{2}$	1.0737 × 10${}^{1}$	−2.835 × 10${}^{2}$	8.9362	−2.609 × 10${}^{2}$	1.3191 × 10${}^{1}$	−2.748 × 10${}^{2}$	9.4686
$f_{13}$	−9.033 × 10${}^{1}$	2.9759 × 10${}^{1}$	−1.571 × 10${}^{2}$	1.3060 × 10${}^{1}$	−1.749 × 10${}^{2}$	1.2035 × 10${}^{1}$	−1.560 × 10${}^{2}$	1.0963 × 10${}^{1}$	−1.654 × 10${}^{2}$	1.4522 × 10${}^{1}$
$f_{14}$	1.2313 × 10${}^{3}$	2.8635 × 10${}^{2}$	4.7548 × 10${}^{2}$	1.9886 × 10${}^{2}$	3.5539 × 10${}^{2}$	2.2408 × 10${}^{2}$	8.4257 × 10${}^{2}$	3.0412 × 10${}^{2}$	2.1266 × 10${}^{2}$	1.6465 × 10${}^{2}$
$f_{15}$	1.3584 × 10${}^{3}$	3.7638 × 10${}^{2}$	9.9741 × 10${}^{2}$	2.9524 × 10${}^{2}$	6.8236 × 10${}^{2}$	3.2280 × 10${}^{2}$	1.2930 × 10${}^{3}$	2.8935 × 10${}^{2}$	1.0151 × 10${}^{3}$	3.0688 × 10${}^{2}$
$f_{16}$	2.0108 × 10${}^{2}$	1.6404 × 10${}^{-1}$	2.0067 × 10${}^{2}$	3.0234 × 10${}^{-1}$	2.0132 × 10${}^{2}$	2.8742 × 10${}^{-1}$	2.0130 × 10${}^{2}$	3.1604 × 10${}^{-1}$	2.0074 × 10${}^{2}$	2.9147 × 10${}^{-1}$
$f_{17}$	4.5492 × 10${}^{2}$	3.7348 × 10${}^{1}$	3.3114 × 10${}^{2}$	1.0234 × 10${}^{1}$	3.2727 × 10${}^{2}$	6.0447	3.5076 × 10${}^{2}$	9.0782	3.2181 × 10${}^{2}$	5.0804
$f_{18}$	5.5529 × 10${}^{2}$	5.2666 × 10${}^{1}$	4.3184 × 10${}^{2}$	1.3605 × 10${}^{1}$	4.3805 × 10${}^{2}$	7.5082	4.5445 × 10${}^{2}$	1.1120 × 10${}^{1}$	4.2980 × 10${}^{2}$	6.6620
$f_{19}$	5.0812 × 10${}^{2}$	3.3968	5.0157 × 10${}^{2}$	7.9477 × 10${}^{-1}$	5.0156 × 10${}^{2}$	6.9150 × 10${}^{-1}$	5.0377 × 10${}^{2}$	1.0163	5.0115 × 10${}^{2}$	4.5671 × 10${}^{-1}$
$f_{20}$	6.0421 × 10${}^{2}$	3.1416 × 10${}^{-1}$	6.0333 × 10${}^{2}$	5.4633 × 10${}^{-1}$	6.0285 × 10${}^{2}$	5.0574 × 10${}^{-1}$	6.0363 × 10${}^{2}$	2.7957 × 10${}^{-1}$	6.0334 × 10${}^{2}$	5.2710 × 10${}^{-1}$
$f_{21}$	1.0551 × 10${}^{3}$	9.4839 × 10${}^{1}$	1.0702 × 10${}^{3}$	7.0293 × 10${}^{1}$	1.1006 × 10${}^{3}$	3.7800 × 10${}^{-1}$	1.1033 × 10${}^{3}$	4.1465 × 10${}^{1}$	1.0836 × 10${}^{3}$	5.2855 × 10${}^{1}$
$f_{22}$	2.4726 × 10${}^{3}$	3.7527 × 10${}^{2}$	1.6389 × 10${}^{3}$	3.0005 × 10${}^{2}$	1.4895 × 10${}^{3}$	3.4980 × 10${}^{2}$	1.9743 × 10${}^{3}$	3.5885 × 10${}^{2}$	1.4281 × 10${}^{3}$	2.2944 × 10${}^{2}$
$f_{23}$	2.5074 × 10${}^{3}$	3.3179 × 10${}^{2}$	2.0434 × 10${}^{3}$	3.4324 × 10${}^{2}$	1.7044 × 10${}^{3}$	4.4608 × 10${}^{2}$	2.1163 × 10${}^{3}$	3.5285 × 10${}^{2}$	2.0472 × 10${}^{3}$	3.6717 × 10${}^{2}$
$f_{24}$	1.2288 × 10${}^{3}$	5.0275	1.2160 × 10${}^{3}$	1.9146 × 10${}^{1}$	1.2115 × 10${}^{3}$	6.2685	1.2202 × 10${}^{3}$	3.6276	1.2178 × 10${}^{3}$	5.0555
$f_{25}$	1.3231 × 10${}^{3}$	4.9373	1.3211 × 10${}^{3}$	5.0100	1.3107 × 10${}^{3}$	5.1177	1.3214 × 10${}^{3}$	2.7814	1.3092 × 10${}^{3}$	2.4628 × 10${}^{1}$
$f_{26}$	1.4041 × 10${}^{3}$	3.8663 × 10${}^{1}$	1.4307 × 10${}^{3}$	6.7151 × 10${}^{1}$	1.3919 × 10${}^{3}$	7.3556 × 10${}^{1}$	1.4005 × 10${}^{3}$	5.5332 × 10${}^{-1}$	1.3861 × 10${}^{3}$	4.8071 × 10${}^{1}$
$f_{27}$	1.9462 × 10${}^{3}$	9.9494 × 10${}^{1}$	1.8572 × 10${}^{3}$	6.9135 × 10${}^{1}$	1.7129 × 10${}^{3}$	8.7656 × 10${}^{1}$	1.8730 × 10${}^{3}$	3.5100 × 10${}^{1}$	1.7836 × 10${}^{3}$	3.9799 × 10${}^{1}$
$f_{28}$	2.3872 × 10${}^{3}$	1.3042 × 10${}^{2}$	1.8734 × 10${}^{3}$	2.0762 × 10${}^{2}$	1.7500 × 10${}^{3}$	1.1019 × 10${}^{2}$	1.9724 × 10${}^{3}$	1.7008 × 10${}^{2}$	1.9112 × 10${}^{3}$	2.4722 × 10${}^{2}$
</=/>	23/0/5		18/0/10		16/0/12		26/1/1		−	

**Table A2:** Comparison of BFGO algorithm and other algorithms on CEC2013 benchmark function under 50D.

$f_{x}$	BA		PSO		GWO		SOA		BFGO	
	Mean	Std	Mean	Std	Mean	Std	Mean	Std	Mean	Std
$f_{1}$	−1.383 × 10${}^{3}$	1.8103	6.6163 × 10${}^{2}$	1.9507 × 10${}^{3}$	1.7472 × 10${}^{3}$	1.8465 × 10${}^{3}$	2.0374 × 10${}^{4}$	5.9767 × 10${}^{3}$	−1.096 × 10${}^{3}$	3.2342 × 10${}^{2}$
$f_{2}$	1.0290 × 10${}^{7}$	2.6234 × 10${}^{6}$	6.9639 × 10${}^{7}$	3.3532 × 10${}^{7}$	5.5385 × 10${}^{7}$	1.6617 × 10${}^{7}$	1.3813 × 10${}^{8}$	4.7348 × 10${}^{7}$	3.9416 × 10${}^{7}$	1.3015 × 10${}^{7}$
$f_{3}$	1.4509 × 10${}^{9}$	1.2689 × 10${}^{9}$	9.6740 × 10${}^{10}$	5.0501 × 10${}^{10}$	1.9432 × 10${}^{10}$	5.8728 × 10${}^{9}$	4.9613 × 10${}^{10}$	8.9406 × 10${}^{9}$	1.9021 × 10${}^{10}$	8.0204 × 10${}^{9}$
$f_{4}$	1.0877 × 10${}^{5}$	2.8748 × 10${}^{4}$	5.2031 × 10${}^{4}$	1.5635 × 10${}^{4}$	5.6337 × 10${}^{4}$	9.5518 × 10${}^{3}$	6.7888 × 10${}^{4}$	8.4160 × 10${}^{3}$	5.6341 × 10${}^{4}$	7.4857 × 10${}^{3}$
$f_{5}$	−9.952 × 10${}^{2}$	3.9260 × 10${}^{-1}$	1.2258 × 10${}^{3}$	1.9889 × 10${}^{3}$	−8.676	3.3203 × 10${}^{2}$	2.4606 × 10${}^{3}$	1.4971 × 10${}^{3}$	−7.340 × 10${}^{2}$	5.6941 × 10${}^{1}$
$f_{6}$	−8.238 × 10${}^{2}$	3.8683 × 10${}^{1}$	−5.831 × 10${}^{2}$	1.3695 × 10${}^{2}$	−6.070 × 10${}^{2}$	7.5124 × 10${}^{1}$	2.5303 × 10${}^{2}$	3.1369 × 10${}^{2}$	−7.070 × 10${}^{2}$	5.2098 × 10${}^{1}$
$f_{7}$	5.9940 × 10${}^{3}$	1.1640 × 10${}^{4}$	−5.661 × 10${}^{2}$	9.8719 × 10${}^{1}$	−7.248 × 10${}^{2}$	1.4614 × 10${}^{1}$	-6.668 × 10${}^{2}$	1.6613 × 10${}^{1}$	−6.711 × 10${}^{2}$	2.3436 × 10${}^{1}$
$f_{8}$	−6.787 × 10${}^{2}$	4.6004 × 10${}^{-2}$	−6.788 × 10${}^{2}$	3.2081 × 10${}^{-2}$	−6.787 × 10${}^{2}$	3.9574 × 10${}^{-2}$	-6.788 × 10${}^{2}$	3.9434 × 10${}^{-2}$	−6.788 × 10${}^{2}$	4.6423 × 10${}^{-2}$
$f_{9}$	−5.325 × 10${}^{2}$	5.0558	−5.386 × 10${}^{2}$	4.5053	−5.584 × 10${}^{2}$	3.5605	-5.364 × 10${}^{2}$	5.2949	−5.384 × 10${}^{2}$	4.7204
$f_{10}$	−4.931 × 10${}^{2}$	1.3669	5.8897 × 10${}^{2}$	5.6839 × 10${}^{2}$	2.3073 × 10${}^{2}$	2.7002 × 10${}^{2}$	1.8301 × 10${}^{3}$	5.3997 × 10${}^{2}$	−3.022 × 10${}^{2}$	4.9227 × 10${}^{1}$
$f_{11}$	7.0216 × 10${}^{2}$	1.6976 × 10${}^{2}$	2.4000 × 10${}^{2}$	7.7858 × 10${}^{1}$	−1.741 × 10${}^{2}$	3.3734 × 10${}^{1}$	2.0925 × 10${}^{2}$	6.7253 × 10${}^{1}$	8.7185 × 10${}^{1}$	1.0942 × 10${}^{2}$
$f_{12}$	8.5553 × 10${}^{2}$	1.8454 × 10${}^{2}$	3.6411 × 10${}^{2}$	1.1549 × 10${}^{2}$	−2.296 × 10${}^{1}$	7.7324 × 10${}^{1}$	3.1815 × 10${}^{2}$	7.8326 × 10${}^{1}$	2.4418 × 10${}^{2}$	1.4352 × 10${}^{2}$
$f_{13}$	1.1097 × 10${}^{3}$	1.5346 × 10${}^{2}$	5.3618 × 10${}^{2}$	1.0069 × 10${}^{2}$	2.1441 × 10${}^{2}$	9.1499 × 10${}^{1}$	4.9592 × 10${}^{2}$	5.3566 × 10${}^{1}$	3.6570 × 10${}^{2}$	1.3450 × 10${}^{2}$
$f_{14}$	8.6727 × 10${}^{3}$	6.1909 × 10${}^{2}$	8.9854 × 10${}^{3}$	1.0823 × 10${}^{3}$	6.2244 × 10${}^{3}$	1.4656 × 10${}^{3}$	1.1741 × 10${}^{4}$	9.0535 × 10${}^{2}$	6.6351 × 10${}^{3}$	1.1034 × 10${}^{3}$
$f_{15}$	9.3666 × 10${}^{3}$	1.0530 × 10${}^{3}$	9.9386 × 10${}^{3}$	1.2284 × 10${}^{3}$	1.0322 × 10${}^{4}$	3.5891 × 10${}^{3}$	1.3357 × 10${}^{4}$	9.4375 × 10${}^{2}$	1.0179 × 10${}^{4}$	1.0865 × 10${}^{3}$
$f_{16}$	2.0365 × 10${}^{2}$	2.7577 × 10${}^{-1}$	2.0272 × 10${}^{2}$	6.7189 × 10${}^{-1}$	2.0388 × 10${}^{2}$	3.6358 × 10${}^{-1}$	2.0401 × 10${}^{2}$	5.6717 × 10${}^{-1}$	2.0262 × 10${}^{2}$	5.5048 × 10${}^{-1}$
$f_{17}$	2.6983 × 10${}^{3}$	3.5116 × 10${}^{2}$	1.1792 × 10${}^{3}$	1.6710 × 10${}^{2}$	6.7354 × 10${}^{2}$	8.5138 × 10${}^{1}$	1.2198 × 10${}^{3}$	8.1104 × 10${}^{1}$	8.2842 × 10${}^{2}$	7.8580 × 10${}^{1}$
$f_{18}$	2.7289 × 10${}^{3}$	3.2267 × 10${}^{2}$	1.2596 × 10${}^{3}$	1.7365 × 10${}^{2}$	9.3555 × 10${}^{2}$	5.0618 × 10${}^{1}$	1.3605 × 10${}^{3}$	1.0343 × 10${}^{2}$	9.1815 × 10${}^{2}$	7.0886 × 10${}^{1}$
$f_{19}$	5.7588 × 10${}^{2}$	8.0687	2.6246 × 10${}^{4}$	5.5941 × 10${}^{4}$	1.3409 × 10${}^{3}$	1.1356 × 10${}^{3}$	3.5449 × 10${}^{4}$	4.9445 × 10${}^{4}$	5.5108 × 10${}^{2}$	1.8663 × 10${}^{1}$
$f_{20}$	6.2489 × 10${}^{2}$	2.1231 × 10${}^{-1}$	6.2361 × 10${}^{2}$	8.2959 × 10${}^{-1}$	6.2204 × 10${}^{2}$	9.0362 × 10${}^{-1}$	6.2342 × 10${}^{2}$	7.3848 × 10${}^{-1}$	6.2380 × 10${}^{2}$	6.6203 × 10${}^{-1}$
$f_{21}$	1.6182 × 10${}^{3}$	2.9018 × 10${}^{2}$	1.8543 × 10${}^{3}$	3.8333 × 10${}^{2}$	2.9124 × 10${}^{3}$	6.1084 × 10${}^{2}$	4.4888 × 10${}^{3}$	1.7404 × 10${}^{2}$	1.8462 × 10${}^{3}$	2.6452 × 10${}^{2}$
$f_{22}$	1.2914 × 10${}^{4}$	1.1921 × 10${}^{3}$	1.1209 × 10${}^{4}$	1.0923 × 10${}^{3}$	8.0121 × 10${}^{3}$	1.1179 × 10${}^{3}$	1.3705 × 10${}^{4}$	1.0819 × 10${}^{3}$	9.1425 × 10${}^{3}$	1.4366 × 10${}^{3}$
$f_{23}$	1.2468 × 10${}^{4}$	1.0566 × 10${}^{3}$	1.2326 × 10${}^{4}$	1.3194 × 10${}^{3}$	1.0486 × 10${}^{4}$	2.9243 × 10${}^{3}$	1.4278 × 10${}^{4}$	9.0028 × 10${}^{2}$	1.2177 × 10${}^{4}$	1.7124 × 10${}^{3}$
$f_{24}$	1.4731 × 10${}^{3}$	3.9179 × 10${}^{1}$	1.3883 × 10${}^{3}$	1.4005 × 10${}^{1}$	1.3106 × 10${}^{3}$	1.2248 × 10${}^{1}$	1.3817 × 10${}^{3}$	1.3103 × 10${}^{1}$	1.3708 × 10${}^{3}$	1.3201 × 10${}^{1}$
$f_{25}$	1.4754 × 10${}^{3}$	9.6601	1.5060 × 10${}^{3}$	1.5542 × 10${}^{1}$	1.4574 × 10${}^{3}$	1.1841 × 10${}^{1}$	1.5020 × 10${}^{3}$	1.4279 × 10${}^{1}$	1.4651 × 10${}^{3}$	1.3123 × 10${}^{1}$
$f_{26}$	1.6909 × 10${}^{3}$	1.5062 × 10${}^{1}$	1.6429 × 10${}^{3}$	6.4205 × 10${}^{1}$	1.6000 × 10${}^{3}$	3.8194 × 10${}^{1}$	1.6669 × 10${}^{3}$	1.1536 × 10${}^{1}$	1.6590 × 10${}^{3}$	1.1516 × 10${}^{1}$
$f_{27}$	3.8321 × 10${}^{3}$	2.0766 × 10${}^{2}$	3.2727 × 10${}^{3}$	1.0808 × 10${}^{2}$	2.6967 × 10${}^{3}$	1.2526 × 10${}^{2}$	3.3107 × 10${}^{3}$	1.4174 × 10${}^{2}$	3.3245 × 10${}^{3}$	1.3656 × 10${}^{2}$
$f_{28}$	1.0205 × 10${}^{4}$	1.0577 × 10${}^{3}$	5.2892 × 10${}^{3}$	2.1351 × 10${}^{3}$	2.9264 × 10${}^{3}$	1.1033 × 10${}^{3}$	5.9023 × 10${}^{3}$	9.7219 × 10${}^{2}$	4.3700 × 10${}^{3}$	2.3070 × 10${}^{3}$
</=/>	20/0/8		21/1/6		13/0/17		25/1/2		−	

**Table A3:** Comparison of BFGO algorithm and other algorithms on CEC2017 benchmark function under 10D.

$f_{x}$	BA		PSO		GWO		SOA		BFGO	
	Mean	Std	Mean	Std	Mean	Std	Mean	Std	Mean	Std
$f_{1}$	4.2293 × 10${}^{5}$	8.5118 × 10${}^{4}$	1.1585 × 10${}^{8}$	3.4578 × 10${}^{8}$	1.9774 × 10${}^{6}$	7.0625 × 10${}^{6}$	3.4701 × 10${}^{8}$	2.3867 × 10${}^{8}$	2.5290 × 10${}^{4}$	1.9533 × 10${}^{4}$
$f_{2}$	2.0057 × 10${}^{2}$	2.1880	1.5486 × 10${}^{7}$	8.4716 × 10${}^{7}$	6.4564 × 10${}^{6}$	1.4605 × 10${}^{7}$	4.0476 × 10${}^{7}$	1.0966 × 10${}^{8}$	4.8763 × 10${}^{3}$	8.9014 × 10${}^{3}$
$f_{3}$	3.0101 × 10${}^{2}$	3.0580 × 10${}^{-1}$	3.0000 × 10${}^{2}$	5.4499 × 10${}^{9}$	1.2754 × 10${}^{3}$	1.2935 × 10${}^{3}$	1.6546 × 10${}^{3}$	1.9474 × 10${}^{3}$	3.0115 × 10${}^{2}$	1.1444
$f_{4}$	4.0219 × 10${}^{2}$	9.6308 × 10${}^{-1}$	4.1182 × 10${}^{2}$	2.2868 × 10${}^{1}$	4.0839 × 10${}^{2}$	4.3872	4.4300 × 10${}^{2}$	2.7242 × 10${}^{1}$	4.0915 × 10${}^{2}$	1.5054 × 10${}^{1}$
$f_{5}$	5.6011 × 10${}^{2}$	1.7863 × 10${}^{1}$	5.2498 × 10${}^{2}$	1.1711 × 10${}^{1}$	5.1177 × 10${}^{2}$	7.4132	5.2212 × 10${}^{2}$	7.0485	5.2665 × 10${}^{2}$	1.1387 × 10${}^{1}$
$f_{6}$	6.3898 × 10${}^{2}$	8.4694	6.0747 × 10${}^{2}$	5.0815	6.0035 × 10${}^{2}$	4.3927 × 10${}^{-1}$	6.0995 × 10${}^{2}$	4.8354	6.0593 × 10${}^{2}$	4.7531
$f_{7}$	8.3113 × 10${}^{2}$	3.3091 × 10${}^{1}$	7.3166 × 10${}^{2}$	1.1849 × 10${}^{1}$	7.2683 × 10${}^{2}$	1.0954 × 10${}^{1}$	7.5549 × 10${}^{2}$	1.3435 × 10${}^{1}$	7.2469 × 10${}^{2}$	6.4241
$f_{8}$	8.4906 × 10${}^{2}$	1.4585 × 10${}^{1}$	8.2121 × 10${}^{2}$	7.4713	8.1326 × 10${}^{2}$	4.4746	8.2367 × 10${}^{2}$	7.2746	8.1852 × 10${}^{2}$	8.1085
$f_{9}$	1.5951 × 10${}^{3}$	3.9579 × 10${}^{2}$	9.2195 × 10${}^{2}$	3.4230 × 10${}^{1}$	9.0607 × 10${}^{2}$	1.7239 × 10${}^{1}$	1.0042 × 10${}^{3}$	6.7349 × 10${}^{1}$	9.0717 × 10${}^{2}$	1.1684 × 10${}^{1}$
$f_{10}$	2.1547 × 10${}^{3}$	3.9922 × 10${}^{2}$	1.7283 × 10${}^{3}$	3.4155 × 10${}^{2}$	1.5018 × 10${}^{3}$	3.3580 × 10${}^{2}$	1.7834 × 10${}^{3}$	2.7596 × 10${}^{2}$	1.9155 × 10${}^{3}$	3.1717 × 10${}^{2}$
$f_{11}$	1.2084 × 10${}^{3}$	7.4265 × 10${}^{1}$	1.1459 × 10${}^{3}$	2.9616 × 10${}^{1}$	1.1213 × 10${}^{3}$	1.1663 × 10${}^{1}$	1.2571 × 10${}^{3}$	7.2155 × 10${}^{1}$	1.1571 × 10${}^{3}$	4.8062 × 10${}^{1}$
$f_{12}$	8.3494 × 10${}^{5}$	6.5698 × 10${}^{5}$	1.0832 × 10${}^{6}$	4.5495 × 10${}^{6}$	7.4796 × 10${}^{5}$	8.3136 × 10${}^{5}$	3.7491 × 10${}^{6}$	4.1740 × 10${}^{6}$	1.3268 × 10${}^{5}$	5.3167 × 10${}^{5}$
$f_{13}$	1.5430 × 10${}^{4}$	1.2175 × 10${}^{4}$	4.2316 × 10${}^{3}$	7.9373 × 10${}^{3}$	1.0964 × 10${}^{4}$	7.0922 × 10${}^{3}$	2.1674 × 10${}^{4}$	2.1984 × 10${}^{4}$	1.5318 × 10${}^{4}$	1.1571 × 10${}^{4}$
$f_{14}$	3.7182 × 10${}^{3}$	3.1837 × 10${}^{3}$	1.4759 × 10${}^{3}$	4.3835 × 10${}^{1}$	2.3807 × 10${}^{3}$	1.5995 × 10${}^{3}$	1.6212 × 10${}^{3}$	2.2511 × 10${}^{2}$	1.4725 × 10${}^{3}$	3.3370 × 10${}^{1}$
$f_{15}$	1.1571 × 10${}^{4}$	9.4442 × 10${}^{3}$	1.6048 × 10${}^{3}$	8.6485 × 10${}^{1}$	4.1679 × 10${}^{3}$	4.0458 × 10${}^{3}$	2.8544 × 10${}^{3}$	1.4257 × 10${}^{3}$	1.7594 × 10${}^{3}$	3.0605 × 10${}^{2}$
$f_{16}$	2.0112 × 10${}^{3}$	1.8594 × 10${}^{2}$	1.7337 × 10${}^{3}$	1.2692 × 10${}^{2}$	1.6868 × 10${}^{3}$	9.7140 × 10${}^{1}$	1.7691 × 10${}^{3}$	1.0430 × 10${}^{2}$	1.7687 × 10${}^{3}$	1.0646 × 10${}^{2}$
$f_{17}$	1.8141 × 10${}^{3}$	6.5555 × 10${}^{1}$	1.7714 × 10${}^{3}$	3.8969 × 10${}^{1}$	1.7621 × 10${}^{3}$	3.6485 × 10${}^{1}$	1.7770 × 10${}^{3}$	3.7898 × 10${}^{1}$	1.7522 × 10${}^{3}$	3.2698 × 10${}^{1}$
$f_{18}$	1.4378 × 10${}^{4}$	1.1066 × 10${}^{4}$	2.2198 × 10${}^{4}$	1.6268 × 10${}^{4}$	2.3569 × 10${}^{4}$	1.5890 × 10${}^{4}$	4.1488 × 10${}^{4}$	1.5111 × 10${}^{4}$	2.0561 × 10${}^{4}$	1.4616 × 10${}^{4}$
$f_{19}$	5.2659 × 10${}^{3}$	3.0924 × 10${}^{3}$	1.9590 × 10${}^{3}$	5.8781 × 10${}^{1}$	7.6128 × 10${}^{3}$	5.9054 × 10${}^{3}$	1.0028 × 10${}^{4}$	7.8380 × 10${}^{3}$	1.9604 × 10${}^{3}$	1.1959 × 10${}^{2}$
$f_{20}$	2.1436 × 10${}^{3}$	7.3136 × 10${}^{1}$	2.0889 × 10${}^{3}$	6.1595 × 10${}^{1}$	2.0752 × 10${}^{3}$	5.9730 × 10${}^{1}$	2.0969 × 10${}^{3}$	5.7679 × 10${}^{1}$	2.0824 × 10${}^{3}$	5.4668 × 10${}^{1}$
$f_{21}$	2.2988 × 10${}^{3}$	7.1270 × 10${}^{1}$	2.3170 × 10${}^{3}$	4.0461 × 10${}^{1}$	2.3018 × 10${}^{3}$	3.4492 × 10${}^{1}$	2.2033 × 10${}^{3}$	1.6705	2.2551 × 10${}^{3}$	6.1269 × 10${}^{1}$
$f_{22}$	2.3110 × 10${}^{3}$	1.4069 × 10${}^{1}$	2.3106 × 10${}^{3}$	1.9266 × 10${}^{1}$	2.3072 × 10${}^{3}$	6.5405	2.8108 × 10${}^{3}$	5.8824 × 10${}^{2}$	2.3044 × 10${}^{3}$	1.1164 × 10${}^{1}$
$f_{23}$	2.6821 × 10${}^{3}$	3.0873 × 10${}^{1}$	2.6371 × 10${}^{3}$	1.2561 × 10${}^{1}$	2.6155 × 10${}^{3}$	8.3301	2.6296 × 10${}^{3}$	1.1686 × 10${}^{1}$	2.6364 × 10${}^{3}$	1.8128 × 10${}^{1}$
$f_{24}$	2.7916 × 10${}^{3}$	1.0332 × 10${}^{2}$	2.7578 × 10${}^{3}$	6.1950 × 10${}^{1}$	2.7430 × 10${}^{3}$	1.1482 × 10${}^{1}$	2.7569 × 10${}^{3}$	1.0514 × 10${}^{1}$	2.7378 × 10${}^{3}$	8.1547 × 10${}^{1}$
$f_{25}$	2.9181 × 10${}^{3}$	6.5990 × 10${}^{1}$	2.9385 × 10${}^{3}$	3.5059 × 10${}^{1}$	2.9338 × 10${}^{3}$	1.6730 × 10${}^{1}$	2.9349 × 10${}^{3}$	1.4572 × 10${}^{1}$	2.9445 × 10${}^{3}$	3.3154 × 10${}^{1}$
$f_{26}$	3.4224 × 10${}^{3}$	3.9858 × 10${}^{2}$	3.0388 × 10${}^{3}$	1.3173 × 10${}^{2}$	2.9442 × 10${}^{3}$	1.9990 × 10${}^{2}$	3.2888 × 10${}^{3}$	4.8915 × 10${}^{2}$	3.0242 × 10${}^{3}$	2.0469 × 10${}^{2}$
$f_{27}$	3.1578 × 10${}^{3}$	3.9004 × 10${}^{1}$	3.1101 × 10${}^{3}$	2.2392 × 10${}^{1}$	3.0979 × 10${}^{3}$	9.0433	3.0940 × 10${}^{3}$	2.5020	3.0910 × 10${}^{3}$	3.3723 × 10${}^{1}$
$f_{28}$	3.2206 × 10${}^{3}$	7.9718 × 10${}^{1}$	3.3422 × 10${}^{3}$	1.3226 × 10${}^{2}$	3.3913 × 10${}^{3}$	5.1933 × 10${}^{1}$	3.2515 × 10${}^{3}$	1.0093 × 10${}^{2}$	3.2425 × 10${}^{3}$	5.8119 × 10${}^{1}$
$f_{29}$	3.3214 × 10${}^{3}$	1.2027 × 10${}^{2}$	3.2174 × 10${}^{3}$	4.7685 × 10${}^{1}$	3.1853 × 10${}^{3}$	4.5780 × 10${}^{1}$	3.1903 × 10${}^{3}$	3.2599 × 10${}^{1}$	3.2467 × 10${}^{3}$	6.0966 × 10${}^{1}$
$f_{30}$	4.4106 × 10${}^{4}$	5.0891 × 10${}^{4}$	1.4518 × 10${}^{6}$	1.7254 × 10${}^{6}$	5.1162 × 10${}^{5}$	6.5062 × 10${}^{5}$	1.2757 × 10${}^{5}$	1.7577 × 10${}^{5}$	1.8622 × 10${}^{4}$	5.3608 × 10${}^{4}$
</=/>	24/0/6		20/0/10		16/0/14		24/0/3		−	

**Table A4:** Comparison of BFGO algorithm and other algorithms on CEC2017 benchmark function under 50D.

$f_{x}$	BA		PSO		GWO		SOA		BFGO	
	Mean	Std	Mean	Std	Mean	Std	Mean	Std	Mean	Std
$f_{1}$	2.5996 × 10${}^{7}$	2.9346 × 10${}^{6}$	5.2824 × 10${}^{9}$	5.7281 × 10${}^{9}$	5.5419 × 10${}^{9}$	2.9747 × 10${}^{9}$	3.3474 × 10${}^{10}$	8.0725 × 10${}^{9}$	7.4546 × 10${}^{8}$	6.4551 × 10${}^{8}$
$f_{2}$	2.0360 × 10${}^{18}$	4.4725 × 10${}^{18}$	5.6044 × 10${}^{71}$	3.0696 × 10${}^{72}$	1.1665 × 10${}^{51}$	4.4867 × 10${}^{51}$	8.9101 × 10${}^{62}$	3.2693 × 10${}^{63}$	4.0636 × 10${}^{51}$	2.2239 × 10${}^{52}$
$f_{3}$	1.4364 × 10${}^{5}$	4.8168 × 10${}^{4}$	8.6954 × 10${}^{4}$	3.4116 × 10${}^{4}$	1.1160 × 10${}^{5}$	1.8904 × 10${}^{4}$	1.2009 × 10${}^{5}$	1.7223 × 10${}^{4}$	9.5425 × 10${}^{4}$	1.7171 × 10${}^{4}$
$f_{4}$	5.5308 × 10${}^{2}$	5.9032 × 10${}^{1}$	1.4340 × 10${}^{3}$	8.0630 × 10${}^{2}$	9.3657 × 10${}^{2}$	2.2268 × 10${}^{2}$	3.4781 × 10${}^{3}$	1.2856 × 10${}^{3}$	7.7404 × 10${}^{2}$	1.0894 × 10${}^{2}$
$f_{5}$	1.1093 × 10${}^{3}$	1.2335 × 10${}^{2}$	8.8483 × 10${}^{2}$	4.7980 × 10${}^{1}$	7.1229 × 10${}^{2}$	4.9750 × 10${}^{1}$	9.1724 × 10${}^{2}$	5.2095 × 10${}^{1}$	8.3948 × 10${}^{2}$	5.5701 × 10${}^{1}$
$f_{6}$	6.9040 × 10${}^{2}$	8.7596	6.5982 × 10${}^{2}$	6.6482	6.1370 × 10${}^{2}$	3.5955	6.6170 × 10${}^{2}$	7.0973	6.5589 × 10${}^{2}$	9.3846
$f_{7}$	2.8054 × 10${}^{3}$	2.4397 × 10${}^{2}$	1.5680 × 10${}^{3}$	1.3551 × 10${}^{2}$	1.0441 × 10${}^{3}$	7.5877 × 10${}^{1}$	1.5758 × 10${}^{3}$	9.8844 × 10${}^{1}$	1.1553 × 10${}^{3}$	8.6659 × 10${}^{1}$
$f_{8}$	1.4327 × 10${}^{3}$	9.8047 × 10${}^{1}$	1.1784 × 10${}^{3}$	5.9460 × 10${}^{1}$	1.0033 × 10${}^{3}$	3.1279 × 10${}^{1}$	1.2231 × 10${}^{3}$	3.5670 × 10${}^{1}$	1.1470 × 10${}^{3}$	5.7759 × 10${}^{1}$
$f_{9}$	3.6998 × 10${}^{4}$	8.4008 × 10${}^{3}$	1.3080 × 10${}^{4}$	2.7610 × 10${}^{3}$	5.4608 × 10${}^{3}$	2.4573 × 10${}^{3}$	2.1399 × 10${}^{4}$	4.4859 × 10${}^{3}$	1.2223 × 10${}^{4}$	3.5224 × 10${}^{3}$
$f_{10}$	9.1541 × 10${}^{3}$	9.9009 × 10${}^{2}$	9.6018 × 10${}^{3}$	1.0159 × 10${}^{3}$	7.9802 × 10${}^{3}$	2.5234 × 10${}^{3}$	1.2672 × 10${}^{4}$	1.3785 × 10${}^{3}$	8.9844 × 10${}^{3}$	1.2649 × 10${}^{3}$
$f_{11}$	1.4787 × 10${}^{3}$	8.7459 × 10${}^{1}$	1.8884 × 10${}^{3}$	4.9847 × 10${}^{2}$	3.7474 × 10${}^{3}$	1.5741 × 10${}^{3}$	6.5202 × 10${}^{3}$	2.1345 × 10${}^{3}$	1.8712 × 10${}^{3}$	2.3450 × 10${}^{2}$
$f_{12}$	7.0553 × 10${}^{7}$	3.5334 × 10${}^{7}$	2.3810 × 10${}^{9}$	2.6378 × 10${}^{9}$	7.3794 × 10${}^{8}$	9.1607 × 10${}^{8}$	5.3287 × 10${}^{9}$	2.1329 × 10${}^{9}$	2.2972 × 10${}^{8}$	2.7394 × 10${}^{8}$
$f_{13}$	2.4616 × 10${}^{6}$	4.9332 × 10${}^{5}$	1.3173 × 10${}^{9}$	2.3459 × 10${}^{9}$	1.2681 × 10${}^{8}$	1.3877 × 10${}^{8}$	1.2046 × 10${}^{9}$	1.3709 × 10${}^{9}$	5.1631 × 10${}^{5}$	3.7632 × 10${}^{5}$
$f_{14}$	1.4477 × 10${}^{5}$	7.5507 × 10${}^{4}$	1.1591 × 10${}^{6}$	2.0245 × 10${}^{6}$	6.5289 × 10${}^{5}$	5.0739 × 10${}^{5}$	1.4236 × 10${}^{6}$	9.6042 × 10${}^{5}$	5.6328 × 10${}^{5}$	5.0871 × 10${}^{5}$
$f_{15}$	8.3509 × 10${}^{5}$	1.9403 × 10${}^{5}$	7.4841 × 10${}^{7}$	3.2644 × 10${}^{8}$	1.2036 × 10${}^{7}$	1.4955 × 10${}^{7}$	6.3604 × 10${}^{7}$	5.1677 × 10${}^{7}$	1.0734 × 10${}^{5}$	1.1059 × 10${}^{5}$
$f_{16}$	4.8433 × 10${}^{3}$	6.2042 × 10${}^{2}$	3.9830 × 10${}^{3}$	4.8984 × 10${}^{2}$	3.1431 × 10${}^{3}$	3.9416 × 10${}^{2}$	4.2678 × 10${}^{3}$	4.8146 × 10${}^{2}$	4.0669 × 10${}^{3}$	5.1607 × 10${}^{2}$
$f_{17}$	4.0175 × 10${}^{3}$	4.6202 × 10${}^{2}$	3.8427 × 10${}^{3}$	3.7463 × 10${}^{2}$	2.9043 × 10${}^{3}$	4.2594 × 10${}^{2}$	3.6441 × 10${}^{3}$	3.6229 × 10${}^{2}$	3.5526 × 10${}^{3}$	3.8962 × 10${}^{2}$
$f_{18}$	1.7657 × 10${}^{6}$	1.0588 × 10${}^{6}$	7.3006 × 10${}^{6}$	1.3247 × 10${}^{7}$	5.2799 × 10${}^{6}$	4.1014 × 10${}^{6}$	7.5737 × 10${}^{6}$	6.1990 × 10${}^{6}$	3.0399 × 10${}^{6}$	2.2408 × 10${}^{6}$
$f_{19}$	5.0522 × 10${}^{6}$	2.5594 × 10${}^{6}$	3.4389 × 10${}^{7}$	1.5369 × 10${}^{8}$	3.1693 × 10${}^{6}$	6.9178 × 10${}^{6}$	6.1074 × 10${}^{7}$	1.8552 × 10${}^{8}$	6.3622 × 10${}^{5}$	9.1839 × 10${}^{5}$
$f_{20}$	3.8384 × 10${}^{3}$	4.0362 × 10${}^{2}$	3.3310 × 10${}^{3}$	3.3364 × 10${}^{2}$	2.9993 × 10${}^{3}$	3.2801 × 10${}^{2}$	3.5795 × 10${}^{3}$	3.7528 × 10${}^{2}$	3.5222 × 10${}^{3}$	3.0390 × 10${}^{2}$
$f_{21}$	3.0118 × 10${}^{3}$	1.0722 × 10${}^{2}$	2.7035 × 10${}^{3}$	6.8215 × 10${}^{1}$	2.5003 × 10${}^{3}$	2.2103 × 10${}^{1}$	2.7309 × 10${}^{3}$	4.5679 × 10${}^{1}$	2.7083 × 10${}^{3}$	7.6226 × 10${}^{1}$
$f_{22}$	1.1129 × 10${}^{4}$	9.9276 × 10${}^{2}$	1.1279 × 10${}^{4}$	1.1399 × 10${}^{3}$	9.9172 × 10${}^{3}$	2.0693 × 10${}^{3}$	1.4385 × 10${}^{4}$	1.0818 × 10${}^{3}$	1.0522 × 10${}^{4}$	7.8315 × 10${}^{2}$
$f_{23}$	4.0802 × 10${}^{3}$	1.7815 × 10${}^{2}$	3.4727 × 10${}^{3}$	1.1112 × 10${}^{2}$	2.9834 × 10${}^{3}$	9.8832 × 10${}^{1}$	3.2475 × 10${}^{3}$	6.7624 × 10${}^{1}$	3.4397 × 10${}^{3}$	2.5472 × 10${}^{2}$
$f_{24}$	4.2572 × 10${}^{3}$	1.2529 × 10${}^{2}$	3.7247 × 10${}^{3}$	1.6005 × 10${}^{2}$	3.1374 × 10${}^{3}$	9.8964 × 10${}^{1}$	3.3203 × 10${}^{3}$	4.5576 × 10${}^{1}$	3.7152 × 10${}^{3}$	1.6440 × 10${}^{2}$
$f_{25}$	2.9957 × 10${}^{3}$	4.4782 × 10${}^{1}$	3.5564 × 10${}^{3}$	6.4807 × 10${}^{2}$	3.4140 × 10${}^{3}$	1.5436 × 10${}^{2}$	5.2906 × 10${}^{3}$	8.1324 × 10${}^{2}$	3.2062 × 10${}^{3}$	8.0942 × 10${}^{1}$
$f_{26}$	1.5330 × 10${}^{4}$	2.7522 × 10${}^{3}$	1.0359 × 10${}^{4}$	1.5911 × 10${}^{3}$	6.3828 × 10${}^{3}$	1.0589 × 10${}^{3}$	8.5240 × 10${}^{3}$	5.4931 × 10${}^{2}$	8.7267 × 10${}^{3}$	3.0592 × 10${}^{3}$
$f_{27}$	4.0892 × 10${}^{3}$	1.0635 × 10${}^{3}$	4.0757 × 10${}^{3}$	2.7373 × 10${}^{2}$	3.5532 × 10${}^{3}$	8.1559 × 10${}^{1}$	3.8512 × 10${}^{3}$	1.8150 × 10${}^{2}$	3.2213 × 10${}^{3}$	5.3457 × 10${}^{1}$
$f_{28}$	3.3000 × 10${}^{3}$	5.7224 × 10${}^{-5}$	5.1795 × 10${}^{3}$	2.2277 × 10${}^{3}$	3.9701 × 10${}^{3}$	3.4515 × 10${}^{2}$	9.0966 × 10${}^{3}$	1.3974 × 10${}^{3}$	3.5687 × 10${}^{3}$	1.8689 × 10${}^{2}$
$f_{29}$	6.2416 × 10${}^{3}$	7.1671 × 10${}^{2}$	6.2585 × 10${}^{3}$	7.2500 × 10${}^{2}$	4.4561 × 10${}^{3}$	3.2569 × 10${}^{2}$	6.5129 × 10${}^{3}$	7.1693 × 10${}^{2}$	6.0243 × 10${}^{3}$	7.8572 × 10${}^{2}$
$f_{30}$	5.4560 × 10${}^{7}$	7.6516 × 10${}^{6}$	5.6907 × 10${}^{7}$	1.1099 × 10${}^{8}$	1.2225 × 10${}^{8}$	4.0766 × 10${}^{7}$	2.2969 × 10${}^{8}$	1.1178 × 10${}^{8}$	1.1685 × 10${}^{7}$	1.3323 × 10${}^{7}$
</=/>	21/0/9		26/0/2		14/0/16		27/0/3		−	
